# Bioglass could increase cell membrane fluidity with ion products to develop its bioactivity

**DOI:** 10.1111/cpr.12906

**Published:** 2020-10-11

**Authors:** Longxin Yan, Haiyan Li, Weiliang Xia

**Affiliations:** ^1^ School of Biomedical Engineering and Med‐X Research Institute Shanghai Jiao Tong University Shanghai China

**Keywords:** Bioglass, cell activity, membrane fluidity, silicon

## Abstract

**Objectives:**

Silicate bioactive glass (BG) has been widely demonstrated to stimulate both of the hard and soft tissue regeneration, in which ion products released from BG play important roles. However, the mechanism by which ion products act on cells on cells is unclear.

**Materials and methods:**

Human umbilical vein endothelial cells and human bone marrow stromal cells were used in this study. Fluorescence recovery after photobleaching and generalized polarization was used to characterize changes in cell membrane fluidity. Migration, differentiation and apoptosis experiments were carried out. RNA and protein chip were detected. The signal cascade is simulated to evaluate the effect of increased cell membrane fluidity on signal transduction.

**Results:**

We have demonstrated that ion products released from BG could effectively enhance cell membrane fluidity in a direct and physical way, and Si ions may play a major role. Bioactivities of BG ion products on cells, such as migration and differentiation, were regulated by membrane fluidity. Furthermore, we have proved that BG ion products could promote apoptosis of injured cells based on our conclusion that BG ion products increased membrane fluidity.

**Conclusions:**

This study proved that BG ion products could develop its bioactivity on cells by directly enhancing cell membrane fluidity and subsequently affected cell behaviours, which may provide an explanation for the general bioactivities of silicate material.

## Graphical abstract

Ion products released from 45S5 Bioglass can significantly enhance cell membrane fluidity, showing a faster fluorescence recovery rate in the bleached area. As the fluidity of the cell membrane increases, the signal transduction and cell recognition processes on the cell membrane are accelerated, leading to increased cell migration and differentiation, even membrane‐related apoptosis.

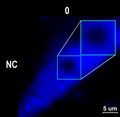

## 1 INTRODUCTION

In tissue engineering, silicate biomaterials are widely reported to participate in tissue regeneration. Bioactive glass (BG), normally composed of SiO_2_, CaO, P_2_O_5_ and Na_2_O, is a typical silicate biomaterial.^1^, ^2^, ^3^ Till now, BG has been proven to promote the regenerations of not only the hard tissue like bone and tooth,^4^ but also soft tissue including skin, blood vessels and myocardium.^1^, ^5^, ^6^, ^7^ Numerous studies have confirmed that ion products of BG could not only affect cell behaviours, such as cell adhesion, proliferation, differentiation, polarization but also enhance intercellular interactions, such as cell‐cell paracrine effects and cell communications through gap junction.^8^, ^9^ Since BG has good solubility, the BG‐containing composites such as polymer/BG composite scaffolds and composite hydrogels could release the same ions and showed the same biological effects as the pure BG.^10^, ^11^, ^12^ Thus, BG and its composites have been widely used in the field of regenerative medicine; a number of clinical products, such as Dermglas^®^, Camgna^®^ and COMBEST^®^, have been developed and commercialized.

Even though many medical products of BG have been developed and applied, the biological effect of BG, especially ionic product, has not been fully studied. It has been widely reported that Si plays critical roles in the biological effects of BG as it is the only component with different concentrations between the culture medium containing ion products of BG and the normal cell culture medium.^1^, ^8^, ^13^, ^14^ But how Si in solution can produce biological effects remains unclear. Although several studies attributed bioactivity of BG to signalling pathways,^13^, ^15^ these conclusions could hardly explain the general biological activities of BG in different types of cells. It is speculated that BG ion products should work in a more primitive way beyond specific signal transduction pathways.

The cell membrane, as the interface between intra‐ and extra‐cellular environments, is the site to receive much of the external information from those including hormone, cytokine, drugs and implanted biomaterials.^16^ Cell membrane fluidity is the basis of cell membrane biological activity. Cells actively change the fluidity of membrane to adapt to different environments and signal transduction needs.^17^ Increased cell membrane fluidity is often coupled with enhanced cell migration, proliferation and differentiation.^18^, ^19^ Furthermore, formation of membrane microdomains, such as lipid rafts and caveolae that play the role of signalling platforms, is also highly related to membrane fluidity.^20^ Membranes, especially changes of membrane fluidity, can produce a wide range of biological effects and are not limited to specific cell types. Thus, we hypothesize that the cell membrane may be one of the main sites where Si exerts its biological activities and Si can optimize the dynamic structure of membrane by affecting the cell membrane fluidity and subsequently enhance cellular signal responses, which contributes to the general biological activities of silicate biomaterials.

Based on these assumptions, herein we investigated the effects of BG ion products released from 45S5 BG on cell membrane fluidity. Fluorescence recovery after photobleaching (FRAP) technology and membrane generalized polarization assay (GP) were used to detect the effects of Si on cell membrane fluidity. By specifically reducing cell membrane fluidity, bioactivity of BG ion products was eliminated. Meanwhile, for injured cells, BG ion products could promote phosphatidylserine flipping of cells and induce the apoptosis of the injured cells. Finally, we simulated changes in signal transduction pathways after the membrane fluidity was increased, indicating that Si had a high impact on overall signal output for cell signal transduction. Overall, we believe that the biological activity of BG ion products may be attributed to the promotion of cell membrane fluidity. This study can not only interpret existing bioactivities of BG materials, but also provide an important theory for expanding the application of BG materials in tissue engineering.

## 2 MATERIALS AND METHODS

### 2.1 Cell isolation and culture

Human umbilical vein endothelial cells (HUVECs) were isolated from human umbilical cord veins using the method reported by Bordenave et al^21^ The isolation of HUVEC was approved by Institutional Review Committee School of Biomedical Engineering and Med‐X Research Institute, Shanghai Jiao Tong University. The isolated cells were cultured in Endothelial culture medium (ECM #1001; ScienCell, Carlsbad, CA, USA) at 37°C in 5% CO_2_. Human bone marrow mesenchyme stem cells (HBMSCs) were purchased from Zhong Qiao Xin Zhou Biotechnology (Shanghai, China) and cultured in Mesenchymal Stem Cell Medium (MSCM# 7501; ScienCell) at 37°C in 5% CO_2_. Only the HUVECs and HBMSCs of early passages (passage 2‐7) were taken for the subsequent experiments. HBMSCs were seeded at a density of 2 × 10^5^ cells per well, and HUVECs were seeded at a density of 3 × 10^5^ cells in 6‐well plates. The seeding density of cells was a quarter of the above‐mentioned values in the FRAP experiment and one half in the GP experiment.

### 2.2 BG ion extracts

BG (45S5 glass) powders with diameter of 5‐20 µm were purchased from Kunshan Chinese Technology New Materials Co., Ltd (Kunshan, China). BG ion extracts were prepared according to the methods reported in literatures adapted from ISO10993‐1 procedures.^22^ Briefly, 1 g of BG powders was soaked in 5 mL of serum‐free ECM and MSCM, respectively. After being incubated for 24 hours in a humidified incubator with 5% CO_2_ at 37°C, the supernatant was then collected and sterilized through a filter (Billerica, MA, USA, Millipore, 0.22 µm). For further use, BG ion extracts were diluted with total ECM (endothelial cell basal medium + 5% foetal bovine serum [FBS] + 1% endothelial cell growth supplement + 1% penicillin‐streptomycin [P/S]) and total MSCM (MSCM + 10% FBS + 1% P/S + 1% l‐glutamine), respectively. The concentrations of Ca, Si and P in the diluted ion extracts were detected by inductively coupled plasma atomic emission spectroscopy (Optima 3000DV; PerkinElmer, San Francisco, CA, USA), and the experiment was repeated three times.

### 2.3 Fluorescence recovery after photobleaching

After the cells were attached on confocal dishes (Shanghai Pumai Biotechnology Co., Ltd, Shanghai, China), lipid dye Laurdan (Thermo Scientific, Waltham, MA, USA) was added to cell culture medium and the cells were cultured for another 3 hours (2 µg/mL). Before FRAP experiments, the Laurdan was washed off with warm PBS (37°C). During the FRAP experiments, an area in the cell membrane for bleaching was selected and laser at a power of 100% generated from a confocal scanning laser microscope was used to bleach the area. After that, the images of the bleached area at different time points were recorded. All the experiments applied the same FRAP setting: 2 frames for pre‐bleach acquisition, 3 s for bleaching and 2 s per frame for 50 times as post‐bleach acquisition. Images were captured, and bleaching process was monitored with TCS SP8 STED 3X system (Leica, Wetzlar, Germany). The fluorescence intensity of bleaching area was analysed, and the data were normalized using a method reported in a previous publication.^23^

### 2.4 Measurement and calculation of GP based on laurdan

Fluorescence spectra of Laurdan are sensitive to the physical state of the surrounding phospholipids gel‐to‐liquid crystalline phase transition, which is associated with the fluidity of cell membranes. The maximum emission peak of Laurdan normally has 50 nm red shift under the increase of membrane fluidity.^24^ After the Lauran in different mixture was excited at 405 nm, the intensities of Laurdan emission at 400‐460 nm (*I*_blue_) and 470‐530 nm (*I*_red_) were measured. Polarization is calculated according to this equation:$\frac{I_{\text{blue}}‐I_{\text{red}}}{I_{\text{blue}}+I_{\text{red}}}$, and GP is the mean value of total polarization. Since GP value is a ratio, it is independent of the local Laurdan concentration. On a scale ranging from −1 to +1, higher GP values correspond to lower membrane fluidity and vice versa. In one experiment, the gain set and offset were consistent such that different groups were comparable. For the GP measurement, cells were cultured on confocal dishes. After the cells were attached, they were cultured with experimental medium. Laurdan was added to culture medium, and cells were incubated with Laurdan for another 3 hours (2 µg/mL) and then washed off before experiment. Fluorescence images of two channels of 400‐460 nm and 470‐530 nm were taken using a confocal microscope (TCS SP5 II; Leica). MATLAB was used to calculate the GP value. Briefly, the captured images were first separated into 4 × 4 PX pieces, and these pieces of *I*_440_ and *I*_490_ were matched by location, respectively. The mean fluorescence intensity per area was used to calculate polarization, and polarization value of each pair of pieces was calculated according to the above equations. Finally, the GP value of one measured area was the mean value of the polarization of each pair of pieces on this area.

### 2.5 Liposome and fluorescence emission spectrum detection

Liposomes were purchased from Xian Ruixi Biological Technology Co., Ltd (Xi'an, China). These liposomes are composed of 100% dipalmitoyl phosphatidylcholine and possess the following properties: diameter of 101.61 nm with a standard deviation of 2.45 and polydispersity index of 0.237 in diameter −28.700 mv with a standard deviation of 0.31 in zeta‐potential. Liposomes were stored at 4°C to maintain their structure. After liposomes (100 µmol/L) were mixed with Laurdan (5 µg/mL) and Si (Si 1/64), the fluorescence emission of the mixture was detected by fluorescence spectrophotometer (F‐2700; Hitachi, Tokyo, Japan) and recorded every 2 hours. For fluorescence scanning parameters, fluorescence excitation wavelength was 390 nm and emission spectrum wavelength was 400‐550 nm.

### 2.6 Assessment of cell migration ability

Wound healing assay and Transwell assay were used to evaluate the migration ability of HUVECs as previously described.^25^ Specific inhibitor of cell membrane fluidity Cholest‐5‐en‐3‐ol (3β)‐, 3‐(hydrogen butanedioate) (CHS) was purchased from Sigma, St. Louis, MO, USA.

### 2.7 Evaluation of osteogenic potential of HBMSCs

Human bone marrow mesenchyme stem cells were seeded at 12‐well culture plates and grew to confluence with MSCM medium. Then, the MSCM medium was removed, and osteogenic induction medium composed of MEM containing 10% FBS, 10 mmol/L β‐glycerophosphate disodium (Sigma, G9422), 0.2 mmol/L ascorbic acid (Sigma, A5960), 0.1 µmol/L dexamethasone (Sigma, D1756)^26^ was added to induce osteogenic differentiation. CHS and Si ion were added in osteogenic induction medium, and HBMSCs were cultured in these media for 7 days. Then, alkaline phosphatase (ALP) kit purchased from Beyotime Biotechnology, Shanghai (P0321), was used to stain the cells to evaluate the ALP protein synthesis in the HBMSCs cultured with different media.

### 2.8 Quantitative real‐time polymerase chain reaction

Quantitative real‐time polymerase chain reaction was conducted as previously described.^27^ β‐actin was used as a housekeeping gene. Primers used in the amplification reaction were as follows: Hsp70: 5′‐TTAGGACAGCTTGTGAGCGG‐3′ (forward), 5′‐CGGCAGTTTCCAAACCCAAG‐3′ (reverse); ALP: 5′‐AGCTCCATCTTCGGGTTGG‐3′ (forward), 5′‐ACGCCTGAGTTGAACACGTA‐3′ (reverse); COLI: 5′‐GGACACAGAGGTTTCAGTGGT‐3′ (forward), 5′‐GCACCATCATTTCCACGAGC‐3′ (reverse); β‐actin: 5′‐GAGCACAGAGCCTCGCC‐3′ (forward), 5′‐TCATCATCCATGGTGAGCTGG‐3′ (reverse).

### 2.9 Evaluation of apoptosis in HUVECs

Healthy HUVECs were seeded at six‐well plates and grew to 70% confluence before the culture media were changed into normal medium (negative control [NC]) or normal medium containing Si (BG extracts diluted to 1/64, 1/128 or 1/256) to investigate the effects of Si on apoptosis of healthy HUVECs. To investigate the effects of Si on apoptosis of injured cells, HUVECs cultured in normal medium or Si 1/128 medium were induced to have apoptosis by different methods. For the DNA damage model, HUVECs were irradiated by UV for 30 minutes at 100 mJ/cm^2^ with a UV lamp. For the ageing model, HUVECs were treated with hydroxyurea at 30 µg/mL for 12 hours (Shanghai, China, Sangon Biotech, A600528). For programmed apoptosis model, HUVECs were treated with TNF‐α, SM‐164 and Z‐VAD‐FMK kits (TSZ, Shanghai, China, Beyotime, C1058) at 1 µL/mL for 12 hours according to the instructions. After induction of apoptosis, cells apoptosis was detected with FITC Annexin V Apoptosis Detection Kit (San Jose, CA, USA, BD Biosciences, 556547). The early or advanced apoptotic cells were distinguished by Annexin‐FITC and PI fluorescence.^27^

### 2.10 Whole gene transcriptome analysis

The whole gene transcriptome microarray data were generated using the Affymetrix Human Genome U133 Plus 2.0 array (Affymetrix, Santa Clara, CA, USA). HBMSCs were cultured with control medium or Si (BG extracts diluted to 1/128) for 3 days. Then, follow the supplier's instructions to collect RNA samples and RNA samples were analysed by Shanghai Biotechnology Corporation, Shanghai, China. Raw data were filtrated for further analysis based on the following criteria: (a) At least one gene data are not A (absent); (b) genetic fold change was more than twice. Selected genes were commented according to GEO, and signalling pathway cluster was done by KEGG.

### 2.11 Protein phosphorylation and signalling pathway screening

The protein expression and phosphorylation data were generated using the PEX100 (Full Moon BioSystems, Inc, Sunnyvale, CA, USA). HBMSCs were cultured with control medium or Si (BG extracts diluted to 1/128) for 7 days. Then wash the cells with pre‐chilled PBS and add the cell lysis solution dedicated to the Full Moon chip to lyse the cells. Add a cell lysis magnetic beads dedicated to the Full Moon chip to each sample, shake at high speed for 1 minute, then stand on ice for 10 minutes and repeat the process 4 times. Centrifuge the sample at 4°C, 15000 *g*, 15 minutes and transfer the supernatant to a new centrifuge tube. Centrifuge the supernatant again and repeat the process four times. After the last centrifugation, transfer the supernatant to a new tube and stored at −20°C before they were subject to the assessment of the protein chip. The chip analysis was done by Wayen Biotechnologies (Shanghai, China), Inc, and signalling pathway enrichment was performed by KEGG.

### 2.12 Simulation of the relationship between cell membrane fluidity and signal transduction

Monte Carlo method is known as the statistical simulation method and is more convenient for discussing the effects of protein movement rate on signal transduction compared with differential model. The classical receptor activation model was chosen for simulation (see Supporting Information, Sup_code). In this model, receptors activated by ligands to form first protein complexes (T1), and the complexes recruit scaffold proteins to form second complexes (T2). Then, the second complexes recruit functional proteins and form third functional complexes (T3) which were able to output biological signals. The third complexes can be depolymerized after collision with inhibitory proteins and each components would re‐engage to signal transduction. For simulation, membrane proteins were regarded as volumetric two‐dimensional particles and protein interactions were represented as particles. The motion of particles obeys the rules of two‐dimensional Brownian motion. The parameters, such as particle density, motion rate, and interaction distance, were employed based on published data.^28^, ^29^ The simulation program was written on MATLAB 2016a.

### 2.13 Statistical analysis

The data were expressed as means ± standard deviation. Statistical significance between groups was calculated using two‐tailed analysis of variance (ANOVA) and performed with a Student's *t* test programme. Statistical significance between two groups was performed with a Student's *t* test programme, and the differences were considered significant when *P* < .05 (*) or *P* < .01 (**). Hsp70 gene expression analysis uses two‐way ANOVA for difference analysis.

## 3 RESULTS

### 3.1 Si in BG ion extracts could increase cell membrane fluidity

Based on the previous research,^30^ BG ion extracts were prepared and diluted to 1/64, 1/128 and 1/256 for cell experiments (Optimal bioactivity range), and Si 1/64, Si 1/128, Si 1/256 were used in the following text and the data to represent the three Si‐containing culture media, respectively. Concentrations of Ca, P and Si were shown in Tables 1 and 2. The concentration of Si in diluted BG ion extracts was significantly higher than that in NC. There is no significant difference in Ca concentration in BG extracts with different dilution ratios, while there is a significant difference in P concentration at higher BG extracts concentrations.

**TABLE 1:** Ion concentration (µg/mL) and pH of HUVEC’s culture medium (n = 3)

	NC	Si 1/64	Si 1/128	Si 1/256
Ca	61.30 ± 0.61	62.58 ± 0.42	61.74 ± 0.41	65.68 ± 1.13
		(*P*> .1)	(*P*> .1)	(*P*> .1)
P	20.71 ± 0.07	23.35 ± 0.15	21.90 ± 0.07	23.08 ± 1.53
		(*P *< .05)	(*P *< .05)	(*P*> .05)
Si	0.53 ± 0.01	1.46 ± 0.01	0.99 ± 0.01	0.70 ± 0.05
		(*P *< .001)	(*P *< .01)	(*P *< .05)
pH	7.57	7.73	7.65	7.57

Abbreviation: HUVEC, human umbilical vein endothelial cell.

**TABLE 2:** Ion concentration (µg/mL) and pH of HBMSC’s culture medium (n = 3)

	NC	Si 1/64	Si 1/128	Si 1/256
Ca	86.57 ± 5.68	83.97 ± 0.32	86.35 ± 4.11	85.75 ± 2.58
		(*P*> .1)	(*P*> .1)	(*P*> .1)
P	25.62 ± 0.33	28.36 ± 0.13	27.45 ± 0.01	26.99 ± 0.04
		(*P *< .05)	(*P*> .05)	(*P*> .1)
Si	0.55 ± 0.01	1.66 ± 0.01	1.13 ± 0.01	0.85 ± 0.01
		(*P *< .0001)	(*P *< .0001)	(*P *< .0001)
pH	7.39	7.58	7.49	7.45

Abbreviation: HBMSC, human bone marrow mesenchyme stem cell.

**FIGURE 1:**
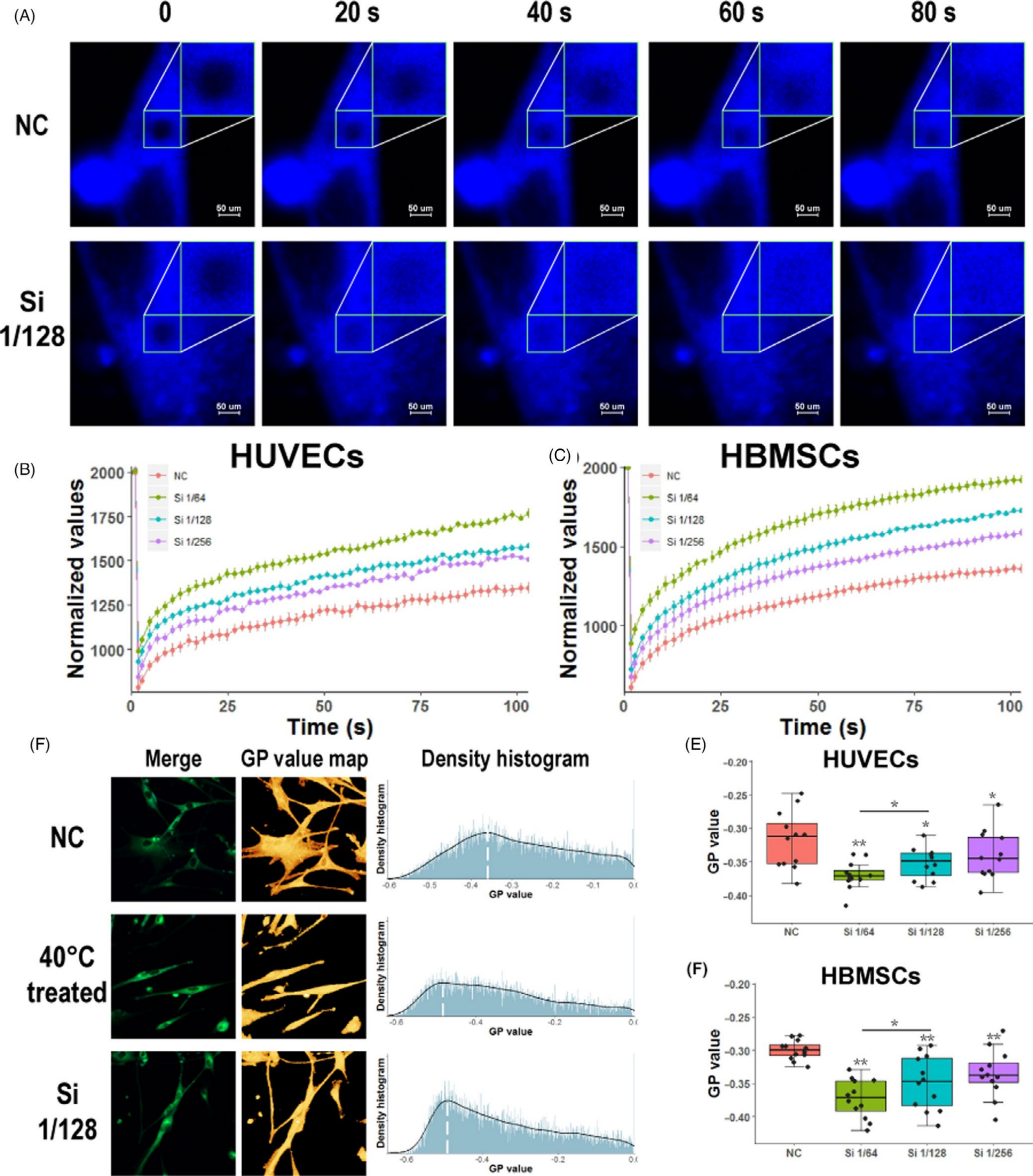
Si of bioactive glass ions extracts could increase cell membrane fluidity. Fluorescence recovery after photobleaching (FRAP) experiment and generalized polarization (GP) value of cell membrane were used to evaluate membrane fluidity. A, Images of HBMSCs in FRAP experiment, which were treated with control medium or Si 1/128 medium. Images were shown at 0, 20, 40, 60, 80 s of whole FRAP experiment. B,C, Normalized fluorescence recovery curve of HUVECs and HBMSCs cultured with different media (n = 9). Fluorescence recovery on membrane was increased with the addition of Si. D, Laurdan fluorescence merged images, polarization value pseudo colour map and polarization value density histogram of HBMSCs cultured with control medium or 40ºC treatment in control medium or Si 1/128 medium. E,F, HUVEC and HBMSCs were treated with different media (n = 12). GP value of membrane was reduced with the addition of Si in the culture medium, which showed the increased membrane fluidity by Si. HBMSC, human bone marrow mesenchyme stem cell; HUVEC, human umbilical vein endothelial cell; NC, negative control

Fluorescence recovery after photobleaching and cell membrane GP were applied to detect the cell membrane fluidity. For FRAP, cells were stained with Laurdan, and subsequently, the fluorescence in a 5 µm‐diameter circular area on cells was bleached and then gradually recovered with time. Fluorescence images of HBMSCs treated with normal or Si 1/128 over time are shown in Figure 1A to represent the FRAP process. Obviously, fluorescence recovery in bleached area was much quicker in HBMSCs treated with Si than that with normal medium. To quantitatively characterize the fluidity of the cell membrane, the fluorescence recovery curves of HUVECs and HBMSCs are shown in Figure 1B,C, respectively, and the fluorescence recovery rate in the bleached area of cells was calculated (see Section 2). The curves showed the intensity value of recovered fluorescence of the bleached membrane areas. Si‐containing media (Si 1/256, Si 1/128 and Si 1/64) could increase fluorescence recovery of the bleached area in HUVECs and HBMSCs as compared to the control medium (Figure 1B,C). Specifically, fluorescence recovery rate in the HUVECs cultured with Si 1/256, Si 1/128 and Si 1/64 was 64.8%, 67.4% and 75.1%, respectively, which were significantly higher than that with control medium (58.5%) (*P* < .01 for all concentrations). Fluorescence recovery rate in the HBMSCs cultured with Si 1/256, Si 1/128 and Si 1/64 was 65.6%, 71.5% and 81.5%, respectively, which were significantly higher than that with control medium (57.2%) (*P* < .01 for all concentrations). In addition, the fluorescence recovery rate in the cells increased with the increasing of Si concentration (*P* < .01 for Si 1/64 and Si 1/128 and *P* < .05 for Si 1/128 and Si 1/256), which suggested that the effects of the Si on cell membrane fluidity were a Si ion concentration‐dependent manner.

Then, GP value was used to reflect the fluidity of whole‐cell membrane. Laurdan fluorescence merged images, polarization value pseudo‐colour map, and polarization value density histogram of HBMSCs with no treatment, incubated at 40°C for 30 minutes (positive control) or treated with Si 1/128, are shown in Figure 1D. According to the polarization value density histograms, treating HBMSCs with Si 1/128 showed the similar effects on the cell membrane polarization to incubating cells at 40°C for 30 minutes, since the cell membrane polarization values in these two groups were much closer to −1 than that in the control group. The GP value of HBMSCs and HUVECs calculated from the whole polarization value is shown in Figure 1E,F, respectively. Specifically, the GP value of HBMSCs cultured with Si 1/256, Si 1/128 and Si 1/64 was −0.335, −0.349 and −0.373, respectively, and that with control medium was −0.300 (n = 12, independent replicates experiment). Similarly, the GP value of HUVECs cultured with Si 1/256, Si 1/128 and Si 1/64 was −0.340, −0.352 and −0.370, respectively, and that with control medium was −0.317 (n = 12). Changes in GP values also indicated that Si could effectively promote changes in cell membrane fluidity in a concentration‐dependent manner (*P* < .01 for all concentrations). Similarly, when we use pure silicate materials (CaSiO_3_) to treat cells, we also find that the fluidity of the cell membrane is increased (see Figure S1). This result may suggest that Si is the main element that affects the fluidity of the cell membrane. We also found that the pure calcium silicate ion extracts were not as effective as the BG material ion extracts, and it may attribute to the synergistic effect of other ions, like P.

### 3.2 Si in BG ion extracts directly and physically interact with the cell membrane

Now that we had confirmed Si could increase the membrane fluidity, and the new question was in which way Si could affect the membrane fluidity: altering membrane lipid component through lipid metabolism pathway or directly interacting with membrane lipids?^31^ For the first hypothesis, Si needs to enter the cells and regulate the expression of metabolism‐related enzymes, which will usually take more than a few hours. On the contrary, by directly interacting with membrane lipids, Si only needs to diffuse to the cell membrane, which will occur with a few minutes. Therefore, we tested the responses of cell membrane fluidity in the presence of Si in a short period of time to test whether the Si directly interact with cell membrane or not.

**FIGURE 2:**
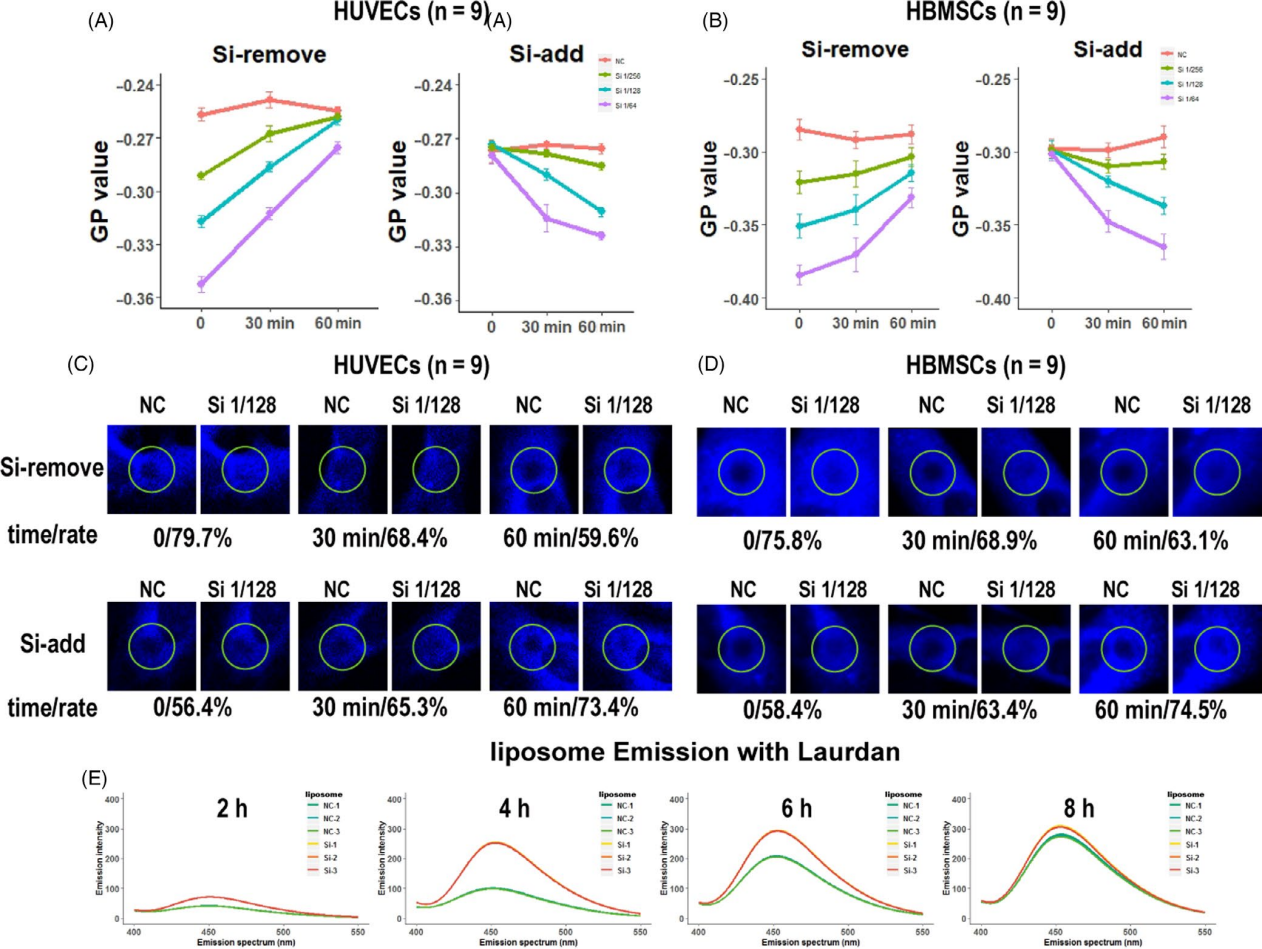
Si interacted with cell membrane to enhance cell membrane fluidity in a direct and physical way. A,B, GP values of cell membranes in HUVECs and HBMSCs were fast and reversibly changed with adding or removing Si (n = 9). C,D, FRAP results indicated that Si had rapid and reversible effects on cell membrane (n = 9). For each pair of graphs, the left side is the fluorescence image of the cells at 0 (bleaching time), and the right side is the fluorescence image of the cells at 50 s. E, The Laurdan fluorescence intensity was increased with time when the cells were cultured with Si 1/128, while the fluorescence spectrum did not change, indicating that the Si did not damage the cell membranes (n = 3). FRAP, fluorescence recovery after photobleaching; GP, generalized polarization; HBMSC, human bone marrow mesenchyme stem cell; HUVEC, human umbilical vein endothelial cell; NC, negative control

Figure 2A,B shows the changes of HUVECs and HBMSCs membrane GP values after removing or adding of Si in culture media within a short time, respectively. After the cells were cultured with Si‐containing medium for 24 hours, the medium was replaced with control medium (Time 0, Si‐remove part in Figure 2A,B). For both of HUVECs and HBMSCs, after the Si was removed for 1 hour, the cell membrane GP value sharply increased and finally recovered to the same level as that in the control group. Then, after the cells rested for several hours, they were cultured with Si‐containing medium again and the GP value was detected in the following 1 hour (S‐add part in Figure 2A,B). Obviously, the cell membrane GP value decreased again.

FRAP was also used to investigate the responses of cell membrane fluidity to the removing or adding of Si in culture media for HUVECs and HBMSCs (Figure 2C,D, respectively). FRAP was applied to cells after the culture condition was changed at 0, 30 and 60 minutes, respectively. Fluorescent images at 0 and 50 s after the cells were bleached were shown to display the fluorescence recovery changes and the fluorescence recovery rates of bleached cells at 50 s were calculated and shown with the images. After the cells were cultured with Si 1/128 medium for 24 hours, the medium was replaced with control medium (top row, Si‐remove part in Figure 2C,D). Overall, after the Si was removed, fluorescence recovery rate of bleached cells decreased with culture time. For HBMSCs, after Si was removed, the fluorescence recovery rate was 75.8% at 0 minute, 68.9% at 30 minutes, and 63.1% at 60 minutes (n = 9), respectively (Figure 2C). Similarly, for HUVECs, after Si was removed, the fluorescence recovery rate was 79.7% at 0 minute, 68.4% at 30 minutes and 59.6% at 60 minutes (n = 9), respectively (Figure 2D). Then, cells rested for several hours and were cultured with Si‐containing medium again (bottom row, Si add part in Figure 2C,D). After the Si was added, fluorescence recovery rate of bleached cells increased with culture time. For HBMSCs, after Si was added again, the fluorescence recovery rate was 58.4% at 0 minute, 63.4% at 30 minutes and 74.5% at 60 minutes (n = 9), respectively (Figure 2C; Figure S2). Similarly, for HUVECs, after Si was added, the fluorescence recovery rate was 56.4% at 0 minute, 65.3% at 30 minutes and 73.4% at 60 minutes (n = 9), respectively (Figure 2D; Figure S3).

The GP value results, together with these FRAP results, suggested that Si caused rapid and reversible changes on cell membrane. To further explore the way through which Si directly interacted with cell membranes, chemical or physical interaction, liposome was used to mimic the cell membrane as it is a typical cellular lipid model for drug and membrane lipid interaction. Laurdan emits fluorescence only after insertion into the lipid membrane, and fluorescence spectra of Laurdan are sensitive to the physical state of the surrounding lipids. When Laurdan is mixed with liposomes, fluorescence intensity of Laurdan is related to the number of Laurdan inserted into liposomes, and fluorescence spectra of Laurdan are related to the lipid structure of liposomes.^32^ The experiments were divided into Si group or NC group according to whether Si was added to the mixture of liposome and Laurdan. As shown in Figures 2E,H, 4H, and 6H after the addition of Laurdan in liposomes, the fluorescence intensity of Si group was much higher as compared to that in the NC group (also shown in Table 3). However, after 8 hours, the fluorescence intensity in all groups reached to same level, which could be the Laurdan loading limit of the liposomes. Besides, the fluorescence spectra of Si group did not show fluorescence emission shifts at all time points as the peak of the fluorescence emission spectrum remained at 450 nm. As the shift of Laurdan spectrum is always accompanied with lipid damage caused by organic solvents or oxidative stress in single component liposomes,^33^ this results indicated that the Si did not damage the lipid component of liposome. Taken together, the interactions of Si with the cell membrane should be physical rather than chemical as the Si only promoted lipid motion without damaging the lipid structure.

**TABLE 3:** The fluorescence emission peak of Laurdan‐mixed liposomes (first line: fluorescence intensity; second line: fluorescence wavelength in nm)

	2 h		4 h		6 h		8 h	
	NC	Si	NC	Si	NC	Si	NC	Si
Group 1	41.8 450 nm	71.4 451 nm	101.5 450.5 nm	254.7 452.5 nm	205.7 452 nm	294.3 452.5 nm	280.9 453.5 nm	309.5 453 nm
Group 2	41.4 450 nm	71.7 450.5 nm	99.5 451 nm	253.7 452.5 nm	206.8 452 nm	293.1 452.5 nm	275.9 453.5 nm	306.0 453 nm
Group 3	41.1 449.5 nm	71.4 450.5 nm	98.9 450.5 nm	252.0 453 nm	208.6 451.5 nm	291.6 452.5 nm	273.0 453.5 nm	304.8 453 nm

### 3.3 Membrane fluidity sensor Hsp70s was highly expressed after stimulation by BG ion extracts

**FIGURE 3:**
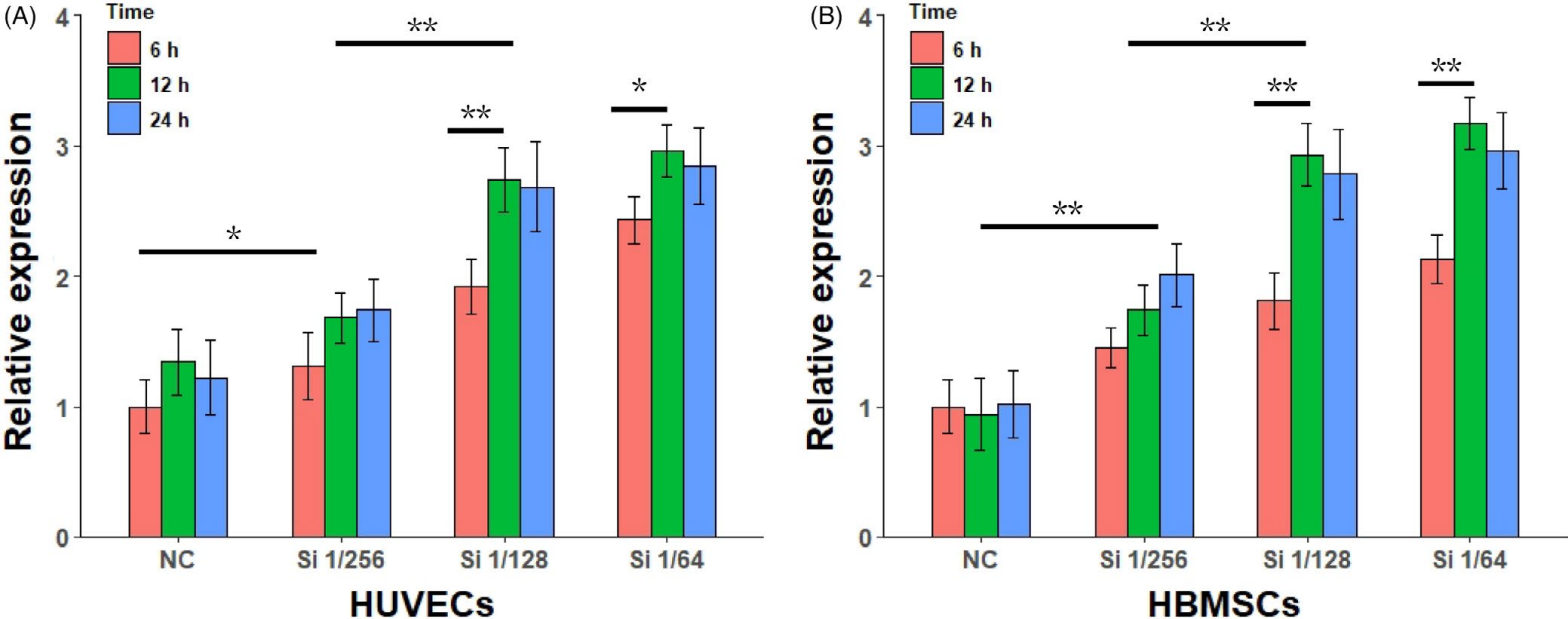
Hsp70 gene expression in HUVECs and HBMSCs after stimulated by Si ion containing BG ion extract (n = 3). *P < .05, and **P < .01 with two‐way analysis of variance. HBMSC, human bone marrow mesenchyme stem cell; HUVEC, human umbilical vein endothelial cell; NC, negative control

As we had demonstrated that BG ion extracts effectively enhanced cell membrane fluidity, we further investigated whether cells have responded to changes in cell membrane fluidity after BG ion extract treatment. Hsp70s expression rises after enhanced cell membrane fluidity and is considered a sensor of cell membrane fluidity.^34^ HUVECs and HBMSCs were treated with Si 1/256, Si 1/128 and Si 1/64. For HUVECs, Hsp70 expressions were significantly induced after Si treatment (Figure 3A, n = 3 for all experiments). Hsp70 gene expression was significantly increased at 6 hours after Si stimulation and further increased at 12 hours, while at 24 hours, Hsp70 gene expression was not significantly different from that at 12 hours. There was no significant difference in Hsp70 expression between Si 1/64 and Si 1/128 treatment. Hsp70 expression on HBMSC also showed the similar results (Figure 3B). These qPCR measurements of Hsp70 expression also revealed that the stimulating effect of BG ion extracts on Hsp70 expression was nearly saturated at Si 1/128 ion concentration. Moreover, Hsp70 expression almost peaked 12 hours after BG ion extracts stimulation. Gene expression of Hsp70 was not completely consistent with the changes in cell membrane fluidity after stimulation with different concentrations of BG ion extracts.

### 3.4 Bioactivity of Si in BG ion extracts was highly related to cell membrane fluidity

**FIGURE 4:**
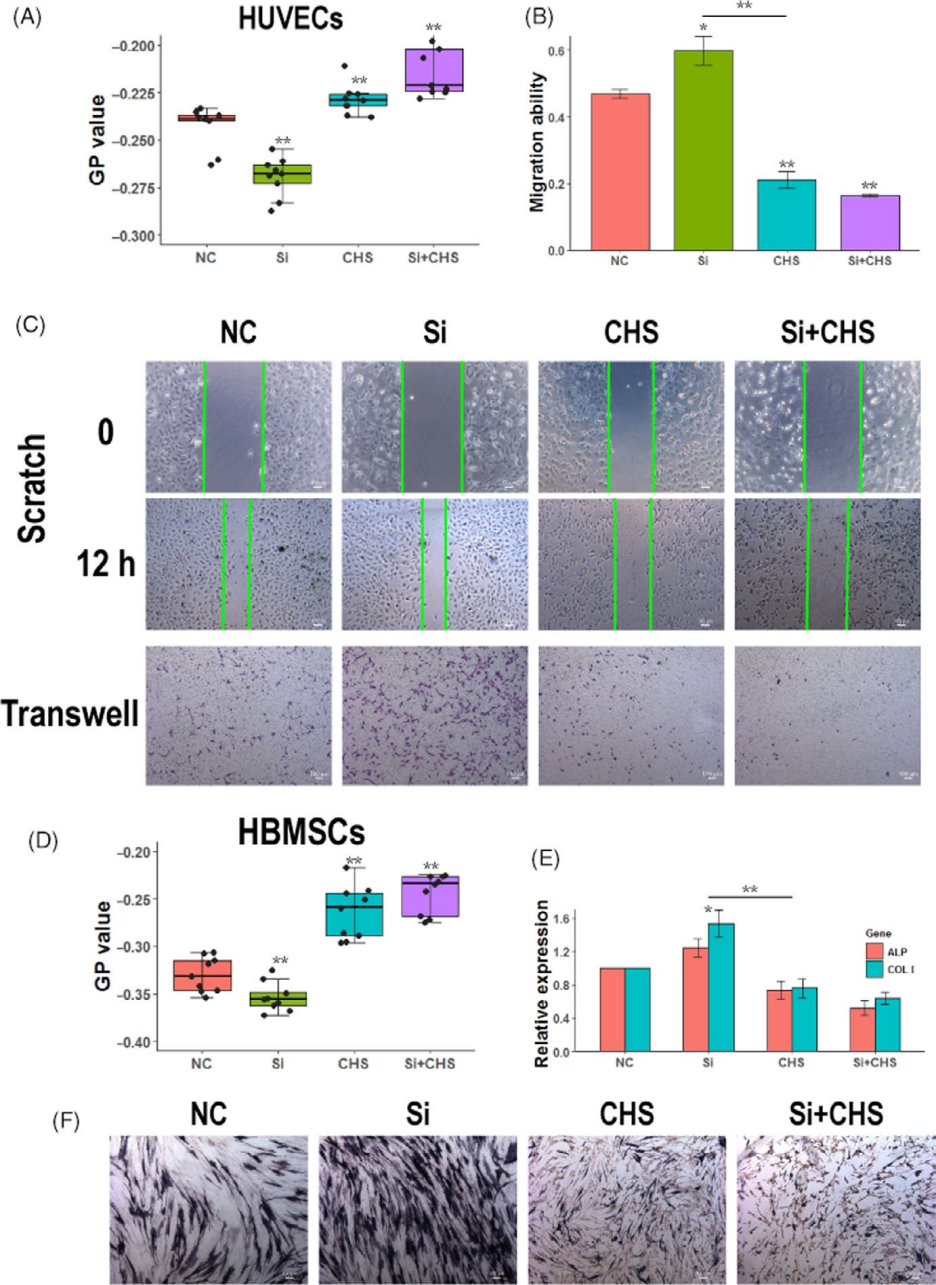
Biological effects of Si on cells were closely related to cell membrane fluidity. A, GP value of HUVECs cultured with Si, CHS and Si + CHS (n = 9). B, Images of wound healing and Transwell assay of HUVECs treated with Si, CHS and Si + CHS (n = 3). C, Quantification of HUVECs migration ability based on wound healing experiments. Si treatment increased HUVEC migration, while CHS treatment inhibited HUVEC migration. Co‐treatment of Si and CHS inhibited HUVEC migration to the same level as CHS treatment. D, GP value of HBMSCs cultured with Si, CHS and Si + CHS (n = 9). E, ALP staining of HBMSCs treated with Si, CHS and Si + CHS (n = 3). F, Gene expressions of ALP and COLI in HBMSCs treated with Si, CHS and Si + CHS (n = 3). Si treatment increased HBMSC osteogenic differentiation while CHS treatment inhibited HBMSC osteogenic differentiation. Co‐treatment of Si and CHS inhibited HBMSC osteogenic differentiation to the same level as CHS treatment. ALP, alkaline phosphatase; CHS, Cholest‐5‐en‐3‐ol (3β)‐, 3‐(hydrogen butanedioate); COLI, collagen type I; GP, generalized polarization; HBMSC, human bone marrow mesenchyme stem cell; HUVEC, human umbilical vein endothelial cell; NC, negative control

To investigate whether biological activities of Si were related to the cell membrane fluidity, cholesterol monoester succinate (CHS) was used to specifically reduce the cell membrane fluidity. As ion extract of BG has been widely reported to promote HUVECs migration and HBMSCs osteogenic differentiation, these cell behaviours were investigated. First, membrane fluidity of cells cultured in control medium (NC), Si‐containing medium (Si 1/128), CHS containing medium (CHS 30 µg/mL), Si and CHS containing medium (Si + CHS) were characterized by GP value (Figure 4A, HUVECs; Figure 4D, HBMSCs). For both of HUVECs and HBMSCs, Si treatment significantly enhanced the cell membrane fluidity while CHS treatment reduced the cell membrane fluidity. Cell membrane fluidity for the Si + CHS group had decreased to the same level as CHS treatment, which may be caused by Si could promote the fusion between CHS and cell membrane.

Wound healing assay and Transwell assay were used to evaluate migration ability of HUVECs cultured with medium of NC, Si, CHS or Si + CHS (Figure 4C). The quantitative results of migration ability of HUVECs were based on wound healing assay (Figure 4B). Compared to that of HUVECs in NC group, the migration ability of HUVECs treated with Si was significantly increased and that of HUVECs treated with CHS was decreased. When HUVECs were co‐treated with Si and CHS, the increased migration ability promoted by Si was completely suppressed to the same level as CHS treatment. For HBMSCs, ALP staining and relative gene expression of ALP and collagen type I (COLI) were used to evaluate HBMSCs osteogenic differentiation. Compared to that of HBMSCs in the NC group, the ALP and COLI expression of HBMSCs treated with Si was significantly increased while that of HBMSCs treated with CHS was decreased. When HBMSCs were co‐treated with Si and CHS, the increased osteogenic differentiation ability promoted by Si was completely suppressed to the same level as CHS treatment (Figure 4E). ALP staining on the HBMSCs showed the same results as relative gene expression (Figure 4F). Taken together, CHS treatment restricted the cell membrane fluidity even in the presence of Si; thus, the stimulatory effects of Si on the migration and differentiation abilities of cells were also eliminated. These results suggested that the stimulatory effects of Si on cell behaviours were highly related to cell membrane fluidity.

### 3.5 Si in BG ion extracts could promote early apoptosis of injured cells

**FIGURE 5:**
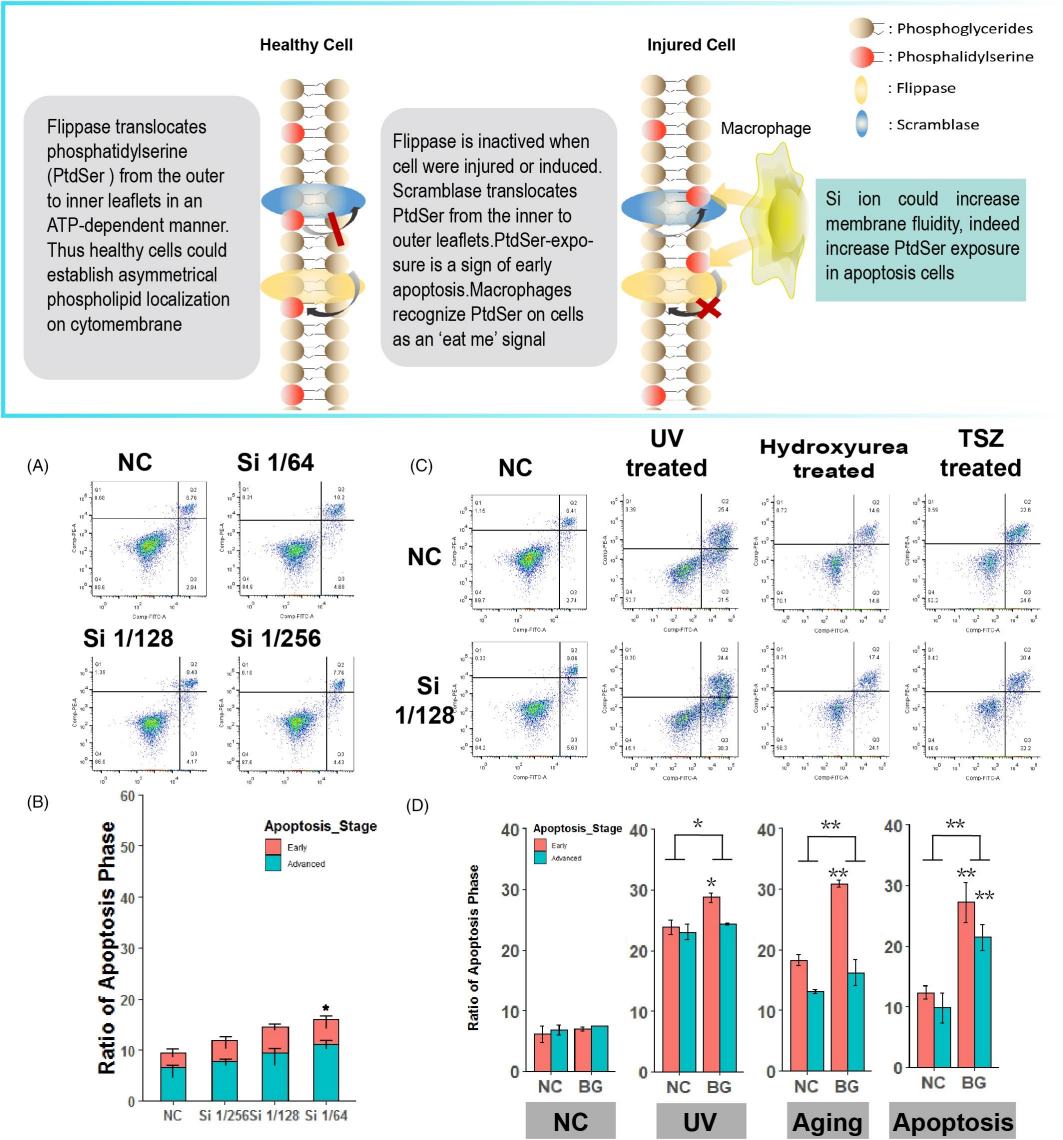
Si could promote apoptosis, especially early apoptosis, of injured HUVECs. A,B, The apoptosis ratio of healthy HUVECs treated with Si 1/64, Si 1/128 or Si 1/256 (n = 3). Si had no significantly affects on healthy HUVECs apoptosis. C,D, The effects of Si on apoptosis ratio of HUVECs in different cell injury models (n = 3). Si significantly increased apoptosis of HUVECs in all apoptotic models. Si significantly increased early apoptosis (marked by phosphatidylserine eversion) of HUVECs. HUVEC, human umbilical vein endothelial cell; NC, negative control

Cell membrane not only works as the site of signal transduction, but also delivers bio‐signals by itself. Phosphatidylserine (PtdSer) flipping from the intracellular leaflet to the outer leaflet was an important starting signal for apoptosis, relaying “eat me” messages to macrophages. As lipid turnover is a form of lipid movement on membrane and is directly related to cell membrane fluidity, we hypothesized that Si could also affect PtdSer flipping and influences apoptosis of cells. First, we investigated the effects of Si on apoptosis in cell cultures. Flow cytometry results and apoptosis histogram of HUVECs treated with Si 1/256, Si 1/128 and Si 1/64 are shown in Figure 5A,B, respectively. Apoptosis ratio was 11.8%, 14.7% and 16.0% in healthy HUVECs treated with Si 1/256, Si 1/128 and Si 1/64, respectively, while that in HUVECs treated with PBS was 9.4%. Statistical analysis indicated that, as compared to HUVECs treated with PBS, only Si 1/64 increased apoptosis of HUVECs (*P* < .05) and Si 1/256, Si 1/128 had no significant influence on the apoptosis of normal cells.

Then, the effects of Si on the apoptosis of injured cells were investigated. HUVECs treated with Si (Si 1/128) or PBS (NC) were induced for apoptosis by either UV, hydroxyurea or TSZ kit (shown in Section 2.9). The flow cytometry as well as apoptosis histogram of HUVECs are shown in Figure 5C,D, respectively. Apoptosis of HUVECs treated with Si was 52.0%, 37.4%, 44.2% in UV‐induced, hydroxyurea‐induced and TSZ‐induced groups, respectively, which was higher than that of HUVECs treated with PBS (44.1%, 28.7%, 35.4%, respectively) (n = 3, *P* < .01). Further analysis indicated that Si treatment mainly enhanced early apoptosis of injured HUVECs. Ratio of early phase apoptosis of HUVECs treated with PBS was 21.7%, 15.2%, 19.9% while that of HUVECs treated with Si was 27.0%, 22.7%, 28.9% in these three injury models, respectively. Therefore, for the increased apoptosis between Si treatment and PBS treatment, early apoptosis contributed 67.1%, 86.2% and 102.3% of the total increased apoptosis in these three injury models. In conclusion, Si treatment could significantly enhance HUVECs apoptosis when the cells were injured, mainly through increasing the ratio of early apoptosis.

### 3.6 Microarray data showed that Si in BG ion extracts did not activate specific signal pathways

**FIGURE 6:**
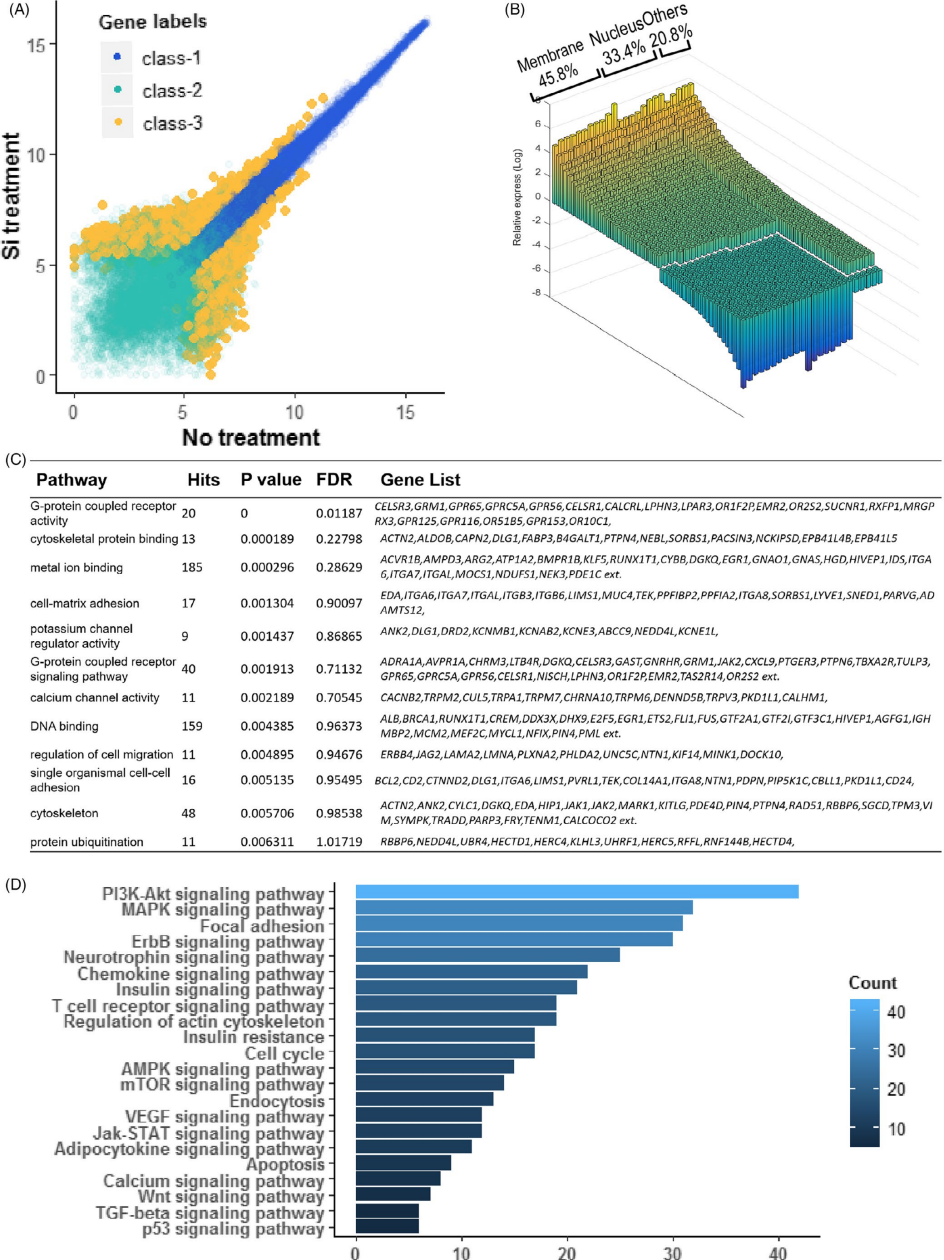
Results obtained from Affymetrix gene expression chip and PEX100 protein activation chip analysis on the HBMSCs cultured with or without Si. A, The scatter plot of gene expression (*X* axis for Si 1/128 treatment, *Y* axis for NC). Differentially expressed genes were shown as class‐3. B, Cellular component distributions of differentially expressed genes according to Gene Ontology. 45.8% of these genes were membrane localized and 33.4% were nucleus localized. Other component were 20.8% only. C, KEGG cluster analysis according to gene expression data. D, KEGG cluster analysis according to protein activations chip. These cluster data showed that numerous base signal pathways were activated, including G protein signal pathways, cytokine pathways, ion channels, PI3K‐IP3 pathways. HBMSC, human bone marrow mesenchyme stem cell; NC, negative control

Gene expression and protein activity of HBMSCs treated with Si from BG ion extracts were analysed by microarray chips. As shown in Figure 6A, various genes in HBMSCs had significant differential expression after stimulation with Si in BG ion extracts (represented as class‐3), which was consistent with the broad bioactivities of Si and BG material. As we sorted genes based on cell location of gene products (Figure 6B), membrane‐related genes, such as ion channels, cell adhesion, bracket of signals, G protein and cytokine receptor, were greatly impacted (n = 557, 45.8%), and the transcription‐related genes were also altered (n = 420, 33.4%). Signalling pathways clustered by KEGG are shown in Figure 6C. These signals were mostly enriched on membrane, such as G protein signal, ion signal, cell adhesion and cytokines signal. Excluding several common signalling pathways, there was no significant difference among these functional signal pathways according to the false discovery rate (FDR) value. Microarray data of protein activity were also clustered by KEGG (Figure 6D). Several common pathways such as PI3K‐Akt signalling pathway and MAPK signalling pathway were enhanced, while other functional pathways showed an average count score according to KEGG cluster. Based on these results, Si treatment could cause a variety of biological activities on HBMSCs as many common signals were activated, while this promotion did not target specific biological process since no functional signal was activated. In addition, in both gene expression and protein activity data, none of metabolism or lipid‐related pathway was significantly changed, which also indicated that Si directly interacted with cell membranes rather than regulating the metabolic pathways in cells.

### 3.7 Simulation of cell membrane fluidity and signal transduction

**FIGURE 7:**
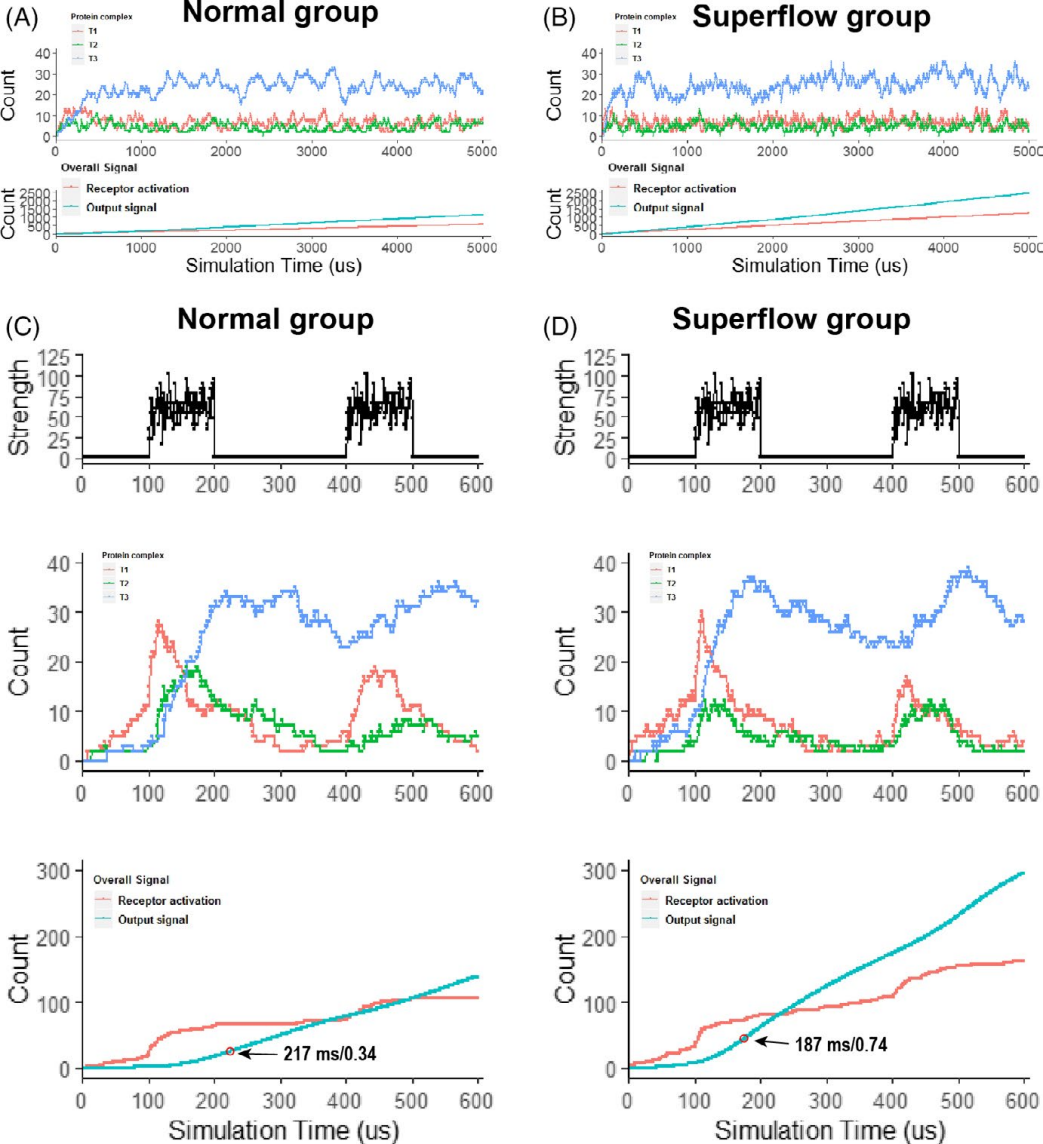
Simulations of the relationship between membrane fluidity and signal transductions. A,B, Effects of membrane fluidity on signal system with resting‐state signal, representing the effects of Si on cells for a long term. For cascade system structures, as T1, T2, T3, increased membrane fluidity had no significant influence on protein activation ratio, which were waved around. However, increased membrane fluidity significantly increased signal output strength, which indicated the long‐term benefit of enhanced membrane fluidity on the signal system. C,D, System responded with step signal. Reactions of signal activations and recovery were more quickly in superfluid group than the normal group. In addition, the secondary response of superfluid group was better than that of the normal group

We have verified the activation of biological signals as caused by changes in cell membrane fluidity, yet it is still not enough to explain the relationship between cell membrane fluidity and biological signal activation in detail. Compared with the single target of common drugs, increased cell membrane fluidity has long‐term, comprehensive and complex influences on signal transduction. Therefore, establishing a mathematical model between cell membrane fluidity and signal transduction is important to better assess the biological activities of Si. Here, we used Monte Carlo simulation to analyse the relationship between signal transduction and cell membrane fluidity (see Supporting Information, Sup_code). To simplify, the three‐level cascade with negative feedback signal modes, same as most receptor tyrosine kinase signalling pathways, was used for simulation. Two types of output data were taken into consideration: protein activation of each transduction point (T1, T2 and T3) and overall signal strength including receptor activation and output signal strength. According to the cell membrane fluidity parameters in the simulation, the simulations were divided into normal group and superfluid group. First, the effects of membrane fluidity on this model with resting‐state signal, representing the effects of Si on cells for a long term, were investigated. Both groups showed periodic oscillations (Figure 7A,B), which also appeared in other simulations and indicated the appropriateness of stimulation.^28^, ^35^ Superfluid group had no more protein activation in each transduction level compared with normal group, while receptor activation and output signal strength were significantly increased in superfluid group. These results indicated that negative feedback system was able to keep the balance of protein activation caused by cell membrane fluidity while increased membrane fluidity could have enhancement on overall signal strength.

Then, the signal system responded to step signal with increased membrane fluidity was investigated (Figure 7C). Step signal was given at 100‐200 ms and 400‐500 ms After the first step when signal appeared at 100 ms, superfluid group responded quicker than normal group (Figure 7C,D), which was proved by a rapid formation of final protein complexes (T3). Furthermore, the time point of maximum growth rate of output signal strength was 187 ms for superfluid group and 217 ms for normal group, and maximum growth rate of output signal strength was 0.74 for superfluid group and 0.34 for normal group, both suggesting that superfluid group had a better signal response than normal group. In addition, when the second step signal was input at 400 ms, superfluid group also showed a better secondary response and more completed signal waveform than normal group according to T3 wave. Based on these results, it could be concluded that Si had a low impact on protein level but had a high impact on overall signal output for cell signal transduction.

## 4 DISCUSSION

In this study, our results demonstrated that BG ion products was very effective in affecting cell membrane fluidity, which may explain the general bio.activities of BG in regulating cell behaviours and tissue regeneration from the point of increased membrane fluidity. We also indicated that effects of BG ion products on cell membrane fluidity were fast and reversible without the involvement of lipid metabolic pathways. Both highly differentiated HUVEC and low differentiated HBMSC show increased cell membrane fluidity after stimulation by BG ion extract, so the effect of BG ion extract on membrane fluidity should be universal. BG ion products have no damage to liposome structure, indicating that the BG ion products directly and physically interacted with cell membrane. For the mechanism of increasing cell membranes fluidity, silicon ions may play a major role, and other ions may play a synergistic role.

The fluidity of the cell membrane depends on the membrane lipids gap, which is caused by the *cis*‐double bond between fatty acids.^31^ Previous studies have shown that hydroxyl is aggressive to double bond; and aluminium ions and ethanol could damage double bond of fatty acids through hydroxyl to alter membrane fluidity.^36^, ^37^ When BG released ion products, Si could form silanol in water solution with weak hydroxyl,^2^ and hydroxyl might help Si approach the gap of double bond and extend the gap, as did some nanoparticles modified with weak hydroxyl.^38^, ^39^ When Si from BG ion extract acted on the membrane, the motion of membrane lipids increased as freer movement space was formed. More research is required to understand the affinity with Si for membrane lipids and their interactive structures.

BG research has always been known for its remarkable effects in in vivo experiments.^40^, ^41^ It may be necessary, but still difficult, to measure cell membrane fluidity in in *vivo* experiments. For instance, how to exclude the influence of fat in tissues requires further study. The other biological effects of BG ion products on cells, such as pH, may also be considered from the perspective of cell membranes. Increased membrane fluidity could directly accelerate the motion of membrane protein and lipids, which facilitates the enzymatic reaction on the membrane.^42^ Previous studies have shown that membrane fluidity is highly related to migration and differentiation of cells.^43^ Once the cell membrane was frozen by CHS, the stimulatory effects of Si on cells migration and differentiation were totally eliminated. If the bioactivity of BG ion products was not related to the cell membrane fluidity, migration and differentiation of culturing cells with a mixture of Si and CHS should not be suppressed to the same level as cells simply cultured with CHS. Thus, cell membrane fluidity played an important role in the stimulatory effects of BG ion products on the cell behaviours. In addition to stimulating the healthy cells to migrate and differentiate, during tissue repairing, removal of injured cells is also important for reducing the diffusion of “death signals” from these injured cells.^44^ In this study, BG ion products could promote apoptosis of injured cells, especially the early apoptosis reflected by PtdSer eversion which is an important apoptosis‐inducing signal and an enzymatic reaction on the membrane. Taken together, Si can not only stimulate the migration and differentiation of healthy cells to but also enhance the apoptosis of injured cells by increasing the cell membrane fluidity, which both are beneficial to stimulating tissue regeneration.

Although our study mainly concerns about the effects of BG ion products on cell membrane fluidity, the interactions between Si and cell membrane will inevitably lead to changes in membrane dynamic feature,^45^ which can significantly affect cell signalling. In this study, many gene products and signalling pathways were up‐regulated and activated by Si, including G protein, hormone and cytokine receptor, scaffold protein, ion channel together with PI3K‐Akt, focal adhesion signal pathway which are all membrane proteins and functionally relate to membrane dynamic feature. For example, amplification of G protein cascade signal specificity occurs in phosphatidylethanolamine formed non‐lamellar phase propensity^46^; voltage‐gated ionic channels are sensitive to the lipid environment.^31^ These lipid domains, such as lipid rafts, caveolae, synaptosomes, receptor clusters, not only enrich membrane protein to particular area, but also regulate signal transduction with lipid environment.^31^ The interaction between Si and membrane may greatly affect the decomposition or recombination of lipid domains and further influence microdomain‐mediated signals transduction.

Simulation models show that enhanced cell membrane fluidity has great impact on cell signal response, with regard to cell signal strength and signal response capabilities. By simply regulating the overall structure of the cell membrane, including membrane microdomain structure, the pattern of signal transduction and drug sensitivity in disease can be altered.^31^, ^42^ Lipid modification could also help drug delivery to correct the abnormality of signal transduction.^42^, ^47^ As we have proved that Si could effectively enhance cell membrane fluidity and silicate biomaterials that can release Si in biological conditions have been widely used in tissue regeneration, it is very promising to apply Si and silicate biomaterials in membrane lipid therapy for chronic diseases.

In conclusion, for the first time, we have confirmed that BG ion products could effectively enhance cell membrane fluidity and the interactions between BG ion products and cell membrane was direct and physical. The stimulatory effects of BG ion products on migration and differentiation of health cells and on apoptosis of injured cells were dependent on the increased of cell membrane fluidity. As BG ion products enhance cell membrane fluidity and the subsequent cell signal transduction in a physical way, applying silicate biomaterials in clinical applications, including tissue regeneration and membrane lipid therapy for different diseases, may be a feasible and efficient strategy.

## CONFLICT OF INTEREST

The authors declare no competing interests.

## AUTHORS' CONTRIBUTIONS

Longxin Yan, Weiliang Xia and Haiyan Li conceived the project. Longxin Yan performed experiment and analysed the data. Longxin Yan, Haiyan Li and Weiliang Xia wrote the manuscript.

## Supporting information

Supplementary Material

## Data Availability

The data that support the findings of this study are available from the corresponding author upon reasonable request.

## References

[1] Yu H , Peng J , Xu Y , Chang J , Li H . Bioglass activated skin tissue engineering constructs for wound healing. ACS Appl Mater Interfaces. 2016;8(1):703‐715. 26684719 10.1021/acsami.5b09853

[2] Fernandes JS , Gentile P , Pires RA , Reis RL , Hatton PV . Multifunctional bioactive glass and glass‐ceramic biomaterials with antibacterial properties for repair and regeneration of bone tissue. Acta Biomater. 2017;59:2‐11. 28676434 10.1016/j.actbio.2017.06.046

[3] Hench LL , Jones JR . Bioactive glasses: frontiers and challenges. Front Bioeng Biotechnol. 2015;3:194. 26649290 10.3389/fbioe.2015.00194 PMC4663244

[4] Dawood AE , Parashos P , Wong RHK , Reynolds EC , Manton DJ . Calcium silicate‐based cements: composition, properties, and clinical applications. J Investig Clin Dent. 2017;8(2):e12195. 10.1111/jicd.12195 26434562

[5] Miguez‐Pacheco V , Hench LL , Boccaccini AR . Bioactive glasses beyond bone and teeth: emerging applications in contact with soft tissues. Acta Biomater. 2015;13:1‐15. 25462853 10.1016/j.actbio.2014.11.004

[6] Barabadi Z , Azami M , Sharifi E , et al. Fabrication of hydrogel based nanocomposite scaffold containing bioactive glass nanoparticles for myocardial tissue engineering. Mater Sci Eng C. 2016;69:1137‐1146. 10.1016/j.msec.2016.08.012 27612811

[7] Hoppe A , Boccaccini AR . Biological impact of bioactive glasses and their dissolution products. Front Oral Biol. 2015;17:22‐32. 26201273 10.1159/000381690

[8] Xu Y , Peng J , Dong X , Xu Y , Li H , Chang J . Combined chemical and structural signals of biomaterials synergistically activate cell‐cell communications for improving tissue regeneration. Acta Biomater. 2017;55:249‐261. 28377306 10.1016/j.actbio.2017.03.056

[9] Ojansivu M , Vanhatupa S , Björkvik L , et al. Bioactive glass ions as strong enhancers of osteogenic differentiation in human adipose stem cells. Acta Biomater. 2015;21:190‐203. 25900445 10.1016/j.actbio.2015.04.017

[10] Gerhardt LC , Boccaccini AR . Bioactive glass and glass‐ceramic scaffolds for bone tissue engineering. Materials. 2010;3(7):3867‐3910. 28883315 10.3390/ma3073867 PMC5445790

[11] Rahaman MN , Day DE , Sonny Bal B , et al. Bioactive glass in tissue engineering. Acta Biomater. 2011;7(6):2355‐2373. 21421084 10.1016/j.actbio.2011.03.016 PMC3085647

[12] Day RM . Bioactive glass stimulates the secretion of angiogenic growth factors and angiogenesis in vitro. Tissue Eng. 2005;11(5–6):768‐777. 15998217 10.1089/ten.2005.11.768

[13] Wang X , Gao L , Han Y , et al. Silicon‐enhanced adipogenesis and angiogenesis for vascularized adipose tissue engineering. Adv Sci. 2018;5(11):1800776. 10.1002/advs.201800776 PMC6247030 30479923

[14] Yi M , Li H , Wang X , et al. Ion therapy: a novel strategy for acute myocardial infarction. Adv Sci. 2019;6(1):1801260. 10.1002/advs.201801260 PMC6325593 30643722

[15] Azevedo MM , Tsigkou O , Nair R , Jones JR , Jell G , Stevens MM . Hypoxia inducible factor‐stabilizing bioactive glasses for directing mesenchymal stem cell behavior. Tissue Eng Part A. 2015;21(1–2):382‐389. 25167933 10.1089/ten.tea.2014.0083 PMC4293089

[16] Jacobson K , Liu P , Lagerholm BC . The lateral organization and mobility of plasma membrane components. Cell. 2019;177(4):806‐819. 31051105 10.1016/j.cell.2019.04.018 PMC6541401

[17] Buda C , Dey I , Balogh N , et al. Structural order of membranes and composition of phospholipids in fish brain cells during thermal acclimatization. Proc Natl Acad Sci U S A. 1994;91(17):8234‐8238. 8058786 10.1073/pnas.91.17.8234 PMC44580

[18] Funaki N , Tanaka J , Kohmoto M , et al. Membrane fluidity correlates with liver cancer cell proliferation and infiltration potential. Oncol Rep. 2001;8(3):527‐532. 11295074 10.3892/or.8.3.527

[19] Noutsi P , Gratton E , Chaieb S . Assessment of membrane fluidity fluctuations during cellular development reveals time and cell type specificity. PLoS One. 2016;11(6):e0158313. 27362860 10.1371/journal.pone.0158313 PMC4928918

[20] Mollinedo F , Gajate C . Lipid rafts as major platforms for signaling regulation in cancer. Adv Biol Regul. 2015;57:130‐146. 25465296 10.1016/j.jbior.2014.10.003

[21] Bordenave L , Baquey CH , Bareille R , et al. Endothelial cell compatibility testing of three different Pellethanes. J Biomed Mater Res. 1993;27(11):1367‐1381. 8262999 10.1002/jbm.820271104

[22] Zhang Y , Niu X , Dong X , Wang Y , Li H . Bioglass enhanced wound healing ability of urine‐derived stem cells through promoting paracrine effects between stem cells and recipient cells. J Tissue Eng Regen Med. 2018;12(3):e1609‐e1622. 29024443 10.1002/term.2587

[23] Lippincott‐Schwartz J , Snapp EL , Phair RD . The development and enhancement of FRAP as a key tool for investigating protein dynamics. Biophys J. 2018;115(7):1146‐1155. 30219286 10.1016/j.bpj.2018.08.007 PMC6170817

[24] Scheinpflug K , Krylova O , Strahl H . Measurement of cell membrane fluidity by Laurdan GP: fluorescence spectroscopy and microscopy. Methods Mol Biol. 2017;1520:159‐174. 27873252 10.1007/978-1-4939-6634-9_10

[25] Kong L , Wu Z , Zhao H , et al. Bioactive injectable hydrogels containing desferrioxamine and bioglass for diabetic wound healing. ACS Appl Mater Interfaces. 2018;10(36):30103‐30114. 30113159 10.1021/acsami.8b09191

[26] Ghali O , Broux O , Falgayrac G , et al. Dexamethasone in osteogenic medium strongly induces adipocyte differentiation of mouse bone marrow stromal cells and increases osteoblast differentiation. BMC Cell Biol. 2015;16:9. 25887471 10.1186/s12860-015-0056-6 PMC4359404

[27] Shao J , Yang X , Liu T , Zhang T , Xie QR , Xia W . Autophagy induction by SIRT6 is involved in oxidative stress‐induced neuronal damage. Protein Cell. 2016;7(4):281‐290. 26983852 10.1007/s13238-016-0257-6 PMC4818841

[28] Fey D , Aksamitiene E , Kiyatkin A , Kholodenko BN . Modeling of receptor tyrosine kinase signaling: computational and experimental protocols. Methods Mol Biol. 2017;1636:417‐453. 28730495 10.1007/978-1-4939-7154-1_27

[29] Saxton MJ . Lateral diffusion in an archipelago. Single‐particle diffusion. Biophys J. 1993;64(6):1766‐1780. 8369407 10.1016/S0006-3495(93)81548-0 PMC1262511

[30] Li H , He J , Yu H , Green CR , Chang J . Bioglass promotes wound healing by affecting gap junction connexin 43 mediated endothelial cell behavior. Biomaterials. 2016;84:64‐75. 26821121 10.1016/j.biomaterials.2016.01.033

[31] Escribá PV , González‐Ros JM , Goñi FM , et al. Membranes: a meeting point for lipids, proteins and therapies. J Cell Mol Med. 2008;12(3):829‐875. 18266954 10.1111/j.1582-4934.2008.00281.x PMC4401130

[32] Moulton ER , Hirsche KJ , Hobbs ML , Schwab JM , Bailey EG , Bell JD . Examining the effects of cholesterol on model membranes at high temperatures: Laurdan and Patman see it differently. Biochim Biophys Acta. 2018;1860(8):1571‐1579. 10.1016/j.bbamem.2018.05.013 29806993

[33] Van der Paal J , Neyts EC , Verlackt CCW , Bogaerts A . Effect of lipid peroxidation on membrane permeability of cancer and normal cells subjected to oxidative stress. Chem Sci. 2016;7(1):489‐498. 28791102 10.1039/c5sc02311d PMC5518669

[34] Armijo G , Okerblom J , Cauvi DM , et al. Interaction of heat shock protein 70 with membranes depends on the lipid environment. Cell Stress Chaperones. 2014;19(6):877‐886. 24789271 10.1007/s12192-014-0511-x PMC4389847

[35] Kageyama R , Shimojo H , Isomura A . Oscillatory control of notch signaling in development. Adv Exp Med Biol. 2018;1066:265‐277. 30030831 10.1007/978-3-319-89512-3_13

[36] Verstraeten SV , Villaverde MS , Oteiza PI . Al(3+)‐mediated changes on membrane fluidity affects the activity of PI‐PLC but not of PLC. Chem Phys Lipid. 2003;122(1–2):159‐163. 10.1016/s0009-3084(02)00192-5 12598047

[37] Nourissat P , Travert M , Chevanne M , et al. Ethanol induces oxidative stress in primary rat hepatocytes through the early involvement of lipid raft clustering. Hepatology. 2008;47(1):59‐70. 18038449 10.1002/hep.21958

[38] Newcomb CJ , Sur S , Lee SS , et al. Supramolecular nanofibers enhance growth factor signaling by increasing lipid raft mobility. Nano Lett. 2016;16(5):3042‐3050. 27070195 10.1021/acs.nanolett.6b00054 PMC4948975

[39] Arribas Perez M , Moriones O , Bastus NG , Puntes V , Nelson AL , Beales PA . Mechanomodulation of lipid membranes by weakly aggregating silver nanoparticles. Biochemistry. 2019;58(47):4761‐4773. 31508939 10.1021/acs.biochem.9b00390

[40] Scholey DV , Belton DJ , Burton EJ , Perry CC . Bioavailability of a novel form of silicon supplement. Sci Rep. 2018;8(1):17022. 30451899 10.1038/s41598-018-35292-9 PMC6242837

[41] Kargozar S , Baino F , Hamzehlou S , Hill RG , Mozafari M . Bioactive glasses: sprouting angiogenesis in tissue engineering. Trends Biotechnol. 2018;36(4):430‐444. 29397989 10.1016/j.tibtech.2017.12.003

[42] Escribá PV , Busquets X , Inokuchi J‐I , et al. Membrane lipid therapy: Modulation of the cell membrane composition and structure as a molecular base for drug discovery and new disease treatment. Prog Lipid Res. 2015;59:38‐53. 25969421 10.1016/j.plipres.2015.04.003

[43] Rudzka DA , Spennati G , McGarry DJ , et al. Migration through physical constraints is enabled by MAPK‐induced cell softening via actin cytoskeleton re‐organization. J Cell Sci. 2019;132:11. 10.1242/jcs.224071 PMC6589089 31152052

[44] Karppinen SM , Heljasvaara R , Gullberg D , Tasanen K , Pihlajaniemi T . Toward understanding scarless skin wound healing and pathological scarring. F1000Res. 2019;8:787. 10.12688/f1000research.18293.1 PMC6556993 31231509

[45] Tarahovsky YS , Kim YA , Yagolnik EA , Muzafarov EN . Flavonoid‐membrane interactions: involvement of flavonoid‐metal complexes in raft signaling. Biochem Biophys Acta. 2014;1838(5):1235‐1246. 24472512 10.1016/j.bbamem.2014.01.021

[46] Vogler O , Casas J , Capo D , et al. The Gbetagamma dimer drives the interaction of heterotrimeric Gi proteins with nonlamellar membrane structures. J Biol Chem. 2004;279(35):36540‐36545. 15231827 10.1074/jbc.M402061200

[47] Gombos I , Crul T , Piotto S , et al. Membrane‐lipid therapy in operation: the HSP co‐inducer BGP‐15 activates stress signal transduction pathways by remodeling plasma membrane rafts. PLoS One. 2011;6(12):e28818. 22174906 10.1371/journal.pone.0028818 PMC3236211

