# Supplementary material for: Bioglass could increase cell membrane fluidity with ion products to develop its bioactivity
Source: Cell Prolif. 2020 Oct 11;53(11):e12906. doi: 10.1111/cpr.12906 (PMC7653244; doi:10.1111/cpr.12906)
Supplement: Supplementary file 1 — Supplementary Material [file CPR-53-e12906-s001.zip › cpr12906-sup-0001-FigS1-S3.docx]

**Fig.ESI1**


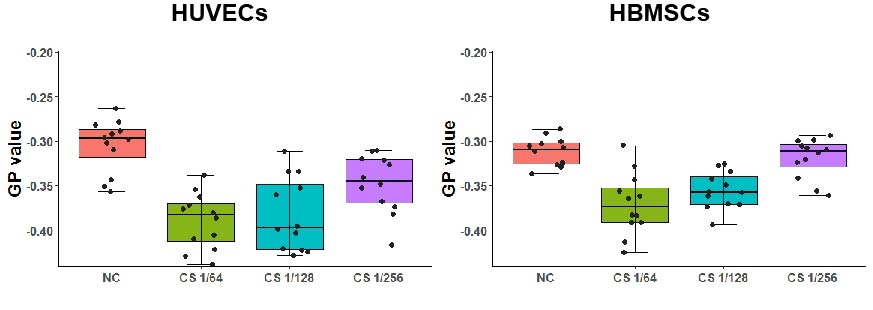


**Fig.ESI1** GP value of HUVECs and HBMSCs treated with Calcium Silicate (CS). The membrane fluidity of HUVECs and HBMSCs increased with the increase of CS extract concentration (n=12).

**Fig.ESI2**

**
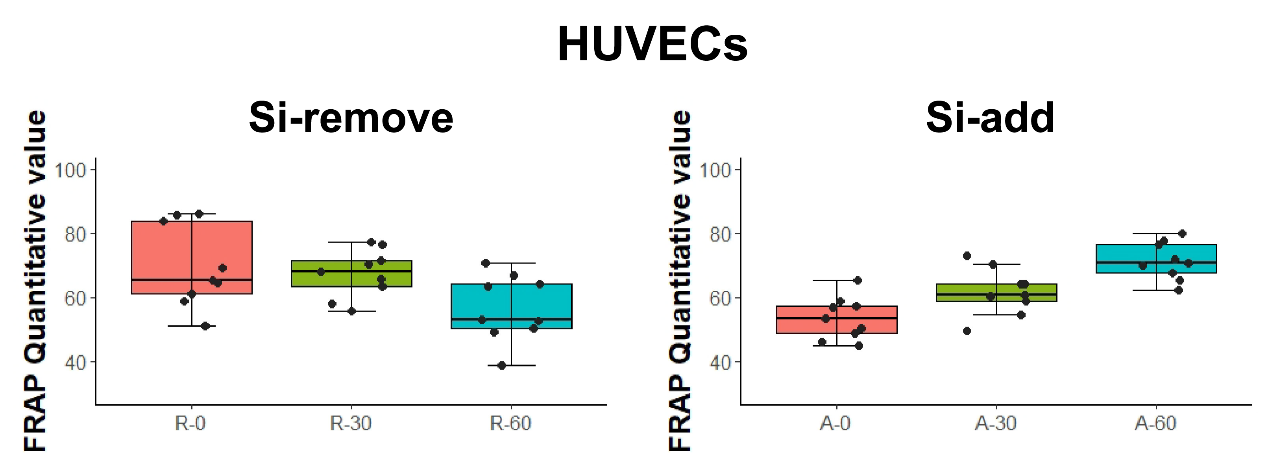
**

**Fig.ESI2** FRAP experiment to investigate the effect of removing and adding Si 1/168 on HUVECs membrane fluidity in a short time (n=9). The quantification of FRAP result is the fluorescence recovery ratio at 50s after bleaching (no unit). R-0, R-30, R-60 represent after Si 1/168 removed at 0, 30 min, 60 min. A-0, A-30, A-60 represent after Si 1/168 added again at 0, 30 min, 60 min.

**Fig.ESI3
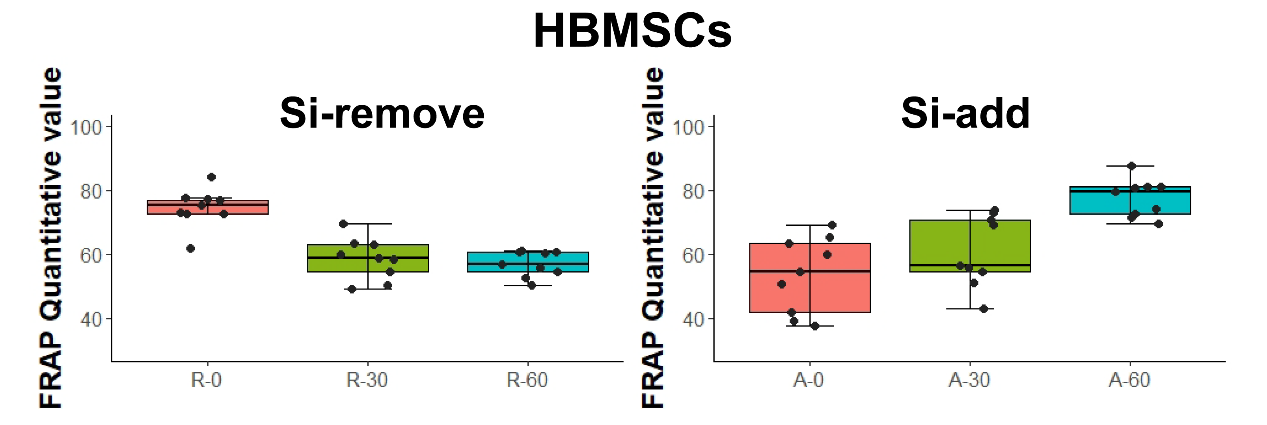
**

**Fig.ESI3** FRAP experiment to investigate the effect of removing and adding Si 1/168 on HBMSCs membrane fluidity in a short time (n=9). The quantification of FRAP result is the fluorescence recovery ratio at 50s after bleaching (no unit). R-0, R-30, R-60 represent after Si 1/168 removed at 0, 30 min, 60 min. A-0, A-30, A-60 represent after Si 1/168 added again at 0, 30 min, 60 min.
