# Supplementary material for: Bioglass could increase cell membrane fluidity with ion products to develop its bioactivity
Source: Cell Prolif. 2020 Oct 11;53(11):e12906. doi: 10.1111/cpr.12906 (PMC7653244; doi:10.1111/cpr.12906)
Supplement: Supplementary file 1 — Supplementary Material [file CPR-53-e12906-s001.zip › cpr12906-sup-0005-Supinfo.docx]

Overview

These programs aim to be a Monte Carlo simulation of cell signal transduction on cell membrane. Basic functions include: Protein_generate (Protein_G, islap), Protein_move(Protein_M) and Protein_combine (Protein_C) were used to simplify the code. For our simulation, a typical three-level cascade signal pathway model with negative feedback regulation was used. These programs only run on MATLAB (2016a).

Program USES

According to Based_functions, generate functions files, respectively. Then add main code to MATLAB and run the main code. The simulation parameters were given in parameter_name.txt for every simulation we did. It could be modified to adapt to simulation conditions.

Based_function

islap.m

function [z]=islap(Conp_free,s)

pg=1;

llgg=length(Conp_free(1,:));

if llgg>2

while pg==1

t=sortrows(Conp_free');

l=length(t(1,:));

for i=1:l-1

if t(i,1)==t(i+1,1)

if t(i,2)==t(i+1,2)

t(i,1)=t(i,1)+round(8*rand(1)-4,1);

t(i,2)=t(i,2)+round(8*rand(1)-4,1);

if t(i,1)>s

t(i,1)=t(i,1)-s;

elseif t(i,1)<0

t(i,1)=t(i,1)+s;

end

if t(i,2)>s

t(i,2)=t(i,2)-s;

elseif t(i,2)<0

t(i,2)=t(i,2)+s;

end

else

1;

end

else

1;

end

end

z=unique(t,'rows');

if length(z)~=length(t)

pg=1;

else

pg=2;

end

end

z=z';

else

z=Conp_free;

end

end

Protein_C.m

function [P,Q,Rection_P]=Protein_C(P,Q,s_s,R_d)

%%

% Ë¼Â·£º

% 1.¶Ô½Ï´óµÄÊý×é½øÐÐÅÅÐò

% 2.¶Ô½ÏÐ¡Êý×éµÄÃ¿Ò»Î»½øÐÐ±È½Ï¡£ÓÃËæ»ú¹ý³ÌÑ¡³öÒ»¸ö´óÖÂµÄÉÏÏÂÏß£¨x²»ÄÜÔ¶ÓÚ10£©£¬È»ºóÔÙÉÏÏÂÏßÄÚ²éÃ¿¸ö¾àÀë£¬Ð¡ÓÚ10¾Í¼ÆÊý¡£

% 3.¶ÔÁ½¸öÊý×éµÄ½áºÏÎ»É¾³ý£¬Êä³öÐÂµÄÁ½¸öÊý×é£¬²¢ÇÒÊä³ö·´Ó¦Êý¡£

if ~isempty(P)&&~isempty(Q)

%%

% A_limx_min=A;A_limx_max=B;A_limy_min=C;A_limy_max=D;

% B_limx_min=a;B_limx_max=b;B_limy_min=c;B_limy_max=d;

%ÅÅÐò£¬¸ù¾ÝX×ø±ê

[P_S,~]=sortrows(P',1);

P=P_S';

Q_L=length(Q(1,:));

Cal_Q=zeros(1,Q_L);

crash_Q=zeros(1,Q_L);flag_p=1;

%%

%ÏÞÖÆÇøÓò

%ÕâÀïµÄË¼Â·ÊÇ¶þ·Ö·¨£¬´ÓX¿ªÊ¼·Ö

for i=1:Q_L

Cal_Q(i)=Q(1,i);P_L=length(P(1,:));

A=1;B=P_L;

if B==0;

break

end

RP_Pick=randi([A,B],1);RP(1)=P(1,RP_Pick);

if Cal_Q(i)-10>0

Mark_x_min=Cal_Q(i)-10;

else

Mark_x_min=0;

end

if Cal_Q(i)+10<s_s

Mark_x_max=Cal_Q(i)+10;

else

Mark_x_max=s_s;

end

%È·¶¨xµÄÉÏÏÂÏß£¬AºÍB

t=RP(1);s=RP_Pick;

while ((t<Mark_x_min||t>Mark_x_max)||((P(1,A)-P(1,s)<-3&&s-A>3)||(P(1,B)-P(1,s)>3&&B-s>3)))&&B-A>2

if t<Mark_x_min

A=s;

s=randi([A,B],1);

t=P(1,s);

else

B=s;

s=randi([A,B],1);

t=P(1,s);

end

end

%°ÑA,BÉèÎªËÑÑ°µÄÉÏÏÂÏß£¬

Pick=(A:B);LP=length(Pick);

P_P=P(:,Pick);

Q_P=Q(:,i);

for kf=1:LP

Distance=norm(P_P(:,kf)-Q_P);

if Distance<R_d %ÅÐ¶ÏÊÇ·ñÐ¡ÓÚ10nm£¬¸øÐ¡ÓÚµÄ¸³1

crash_Q(i)=111;

P_ert(:,flag_p)=P(:,A+kf-1);

P(:,A+kf-1)=[];

Q_ert(:,flag_p)=Q(:,i);

flag_p=flag_p+1;

break;

end

end

end

reset_Q=find(crash_Q==111);

LLL=length(reset_Q);

%%

if LLL~=0

for t=1:LLL

CLEAR_Q=reset_Q(t);

Q(:,CLEAR_Q)=-1;

end

QQ=(Q(1,:)==-1);

Q(:,QQ)=[];

end

%%

if LLL

for p=1:LLL

Rection_P(1,p)=0.5*(P_ert(1,p)+Q_ert(1,p));

Rection_P(2,p)=0.5*(P_ert(2,p)+Q_ert(2,p));

end

else

Rection_P=[];

end

else

P=P;Q=Q;Rection_P=[];

end

end

Protein_G.m

function [P_T]=Protein_G(s_s,num)

s_g=s_s/10;P_T=zeros(2,num);

for i=1:num

x=s_g*rand(1);

y=s_g*rand(1);

x=roundn(x,-1);

y=roundn(y,-1);

w=unidrnd(10);h=unidrnd(10);

P_T(1,i)=x+(w-1)*s_g

P_T(2,i)=y+(h-1)*s_g;

end

P_T=islap(P_T,s_s);

end

Protein_M.m

function [move_location]=Protein_M(location_before,Diffusivity,Step_Time,s_s)

%¶Ôµ°°×µÄÀ©É¢½øÐÐÄ£Äâ£¬²ÎÊýÎªÖ®Ç°µÄÎ»ÖÃºÍÀ©É¢ÏµÊý£»

%µ±À©É¢Ô½¹ý±ß½ç£¬´ÓÁíÒ»±ß·µ»ØÀ´

if ~isempty (location_before)

size_L=length(location_before(1,:));

p_x_ms=randn(1,size_L)*(2*Diffusivity*Step_Time)^0.5;

p_y_ms=randn(1,size_L)*(2*Diffusivity*Step_Time)^0.5;

p_x_ms=roundn(p_x_ms,-1);

p_y_ms=roundn(p_y_ms,-1);

move_location(1,:)=location_before(1,:)+p_x_ms;

move_location(2,:)=location_before(2,:)+p_y_ms;

for i=1:size_L

if move_location(1,i)>s_s

move_location(1,i)=move_location(1,i)-s_s;

elseif move_location(1,i)<0

move_location(1,i)=move_location(1,i)+s_s;

end

if move_location(2,i)>s_s

move_location(2,i)=move_location(2,i)-s_s;

elseif move_location(2,i)<0

move_location(2,i)=move_location(2,i)+s_s;

end

end

move_location=islap(move_location,s_s);

else

move_location=location_before;

end

end

**Main Fuc**

simulation_1_Normal.txt

**s 200**

**s_t 1**

**t 5000**

**R_d 3**

**Mem_flu 3**

**P0 3**

**P1 60**

**P2 100**

**P3 100**

**PB 200**

simulation_1_Normal.m

clc;clear;

Parameter = importdata('simulation_1_Normal.txt');

% s:size of simulation area (shape:square,unit:nm)

% s_t:Simulation step time (us)

% t:Simulation time (us)

% R_d:interac distance of proteins

% Mem_flu:Fluidity of cell membrane (influence interaction times)

% P0:ligand generate num (per step)

% P1:receptor protein num (constant)

% P2:First recruited protein num (constant)

% P3:Second recruited protein num (constant)

% PB:Depolymerization protein num (constant)

s=Parameter.data(1);s_t=Parameter.data(2);t=Parameter.data(3);R_d=Parameter.data(4);Mem_flu=Parameter.data(5);

P0=Parameter.data(6);P1=Parameter.data(7);P2=Parameter.data(8);P3=Parameter.data(9);PB=Parameter.data(10);

%Conp_free repersent legend protein,Conp_1 repersent receptor,Conp_2 repersent First recruited protein,

%Conp_3 repersent Second recruited protein, Conp_break repersent arrestin

%MED-1,2,3 repersent compound 1,2,3

t=t*Mem_flu/3;Conp_1=Protein_G(s,P1);Conp_2=Protein_G(s,P2);Conp_3=Protein_G(s,P3);Conp_break=Protein_G(s,PB);

MED_1=[];MED_2=[];MED_3=[];MED_M_1=[];MED_M_2=[];Conp_repl=[];Count=zeros(1,t);Count_w=zeros(1,t);

%%

for i=1:t

Conp_free=Protein_G(s,P0);

[~,Conp_1,MED_M_1]=Protein_C(Conp_free,Conp_1,s,R_d);

MED_1=[MED_1,MED_M_1];

T1(i)=length(MED_1);

Conp_1=Protein_M(Conp_1,Mem_flu,s_t,s);

if ~isempty (MED_1)

MED_1=Protein_M(MED_1,Mem_flu,s_t,s);

[Conp_2,MED_1,MED_M_2]=Protein_C(Conp_2,MED_1,s,R_d);

Conp_2=Protein_M(Conp_2,Mem_flu,s_t,s);

MED_2=[MED_2,MED_M_2];

T2(i)=length(MED_2);

if ~isempty (MED_2)

MED_2=Protein_M(MED_2,0.8*Mem_flu,s_t,s);

[Conp_3,MED_2,MED_M_3]=Protein_C(Conp_3,MED_2,s,R_d);

MED_3=[MED_3,MED_M_3];

T3(i)=length(MED_3);

if ~isempty (MED_3)

MED_3=Protein_M(MED_3,0.4*Mem_flu,s_t,s);

[~,MED_3,Conp_repl]=Protein_C(Conp_break,MED_3,s,R_d);

Conp_break=Protein_M(Conp_break,Mem_flu,s_t,s);

else

Conp_repl=[];

end

else

T3(i)=length(MED_3);

end

else

T2(i)=length(MED_2);

T3(i)=length(MED_3);

end

if ~isempty(MED_M_1)

Count(i+1)=Count(i)+length(MED_M_1(1,:));

else

Count(i+1)=Count(i);

end

if ~isempty(MED_3)

Count_w(i+1)=Count_w(i)+length(MED_3(1,:));

else

Count_w(i+1)=Count_w(i);

end

% Conp_free=Protein_G(s,50);

%Reactivate the proteins after 50 us

if i<=50

eval(['Sp1_',num2str(i),'=Conp_repl',';']);

eval(['Sp2_',num2str(i),'=Conp_repl',';']);

eval(['Sp3_',num2str(i),'=Conp_repl',';']);

for gn=1:i

eval(['Sp1_',num2str(gn),'=Protein_M(Sp1_',num2str(gn),',2*Mem_flu,s_t,s)',';']);

eval(['Sp2_',num2str(gn),'=Protein_M(Sp1_',num2str(gn),',2*Mem_flu,s_t,s)',';']);

eval(['Sp3_',num2str(gn),'=Protein_M(Sp1_',num2str(gn),',2*Mem_flu,s_t,s)',';']);

end

else

kk=mod(i,50);

if kk==0

kk=50;

end

eval(['Conp_1=[Conp_1,Sp1_',num2str(kk),']',';']);

eval(['Conp_2=[Conp_2,Sp2_',num2str(kk),']',';']);

eval(['Conp_3=[Conp_2,Sp3_',num2str(kk),']',';']);

eval(['Sp1_',num2str(kk),'=Conp_repl',';']);

eval(['Sp2_',num2str(kk),'=Conp_repl',';']);

eval(['Sp3_',num2str(kk),'=Conp_repl',';']);

for gn=1:50

eval(['Sp1_',num2str(gn),'=Protein_M(Sp1_',num2str(gn),',2*Mem_flu,s_t,s)',';']);

eval(['Sp2_',num2str(gn),'=Protein_M(Sp1_',num2str(gn),',2*Mem_flu,s_t,s)',';']);

eval(['Sp3_',num2str(gn),'=Protein_M(Sp1_',num2str(gn),',2*Mem_flu,s_t,s)',';']);

end

end

end

if length(Count)>t

Count(1)=[];

Count_w(1)=[];

end

Count_w_afit=Count_w/100;

%%

%output

% save E:\Desktop\simulation\SIm2\Nor\T1.txt T1 -ascii -tabs

% save E:\Desktop\simulation\SIm2\Nor\T2.txt T2 -ascii -tabs

% save E:\Desktop\simulation\SIm2\Nor\T3.txt T3 -ascii -tabs

% save E:\Desktop\simulation\SIm2\Nor\Count.txt Count -ascii -tabs

% save E:\Desktop\simulation\SIm2\Nor\Count_w_afit.txt Count_w_afit -ascii -tabs

figure

subplot(3,1,1)

plot(T1)

xlabel('Simulation time (us)')

title('Protein complex 1 (T1)')

subplot(3,1,2)

plot(T2)

xlabel('Simulation time (us)')

title('Protein complex 2 (T2)')

subplot(3,1,3)

plot(T3)

xlabel('Simulation time (us)')

title('Protein complex 2 (T3)')

figure

subplot(2,1,1)

plot(Count)

xlabel('Simulation time (us)')

title('Receptor protein usage')

subplot(2,1,2)

plot(Count_w_afit)

xlabel('Simulation time (us)')

title('Signal output strength')

simulation_1_Superfluidity.txt

**s 200**

**s_t 1**

**t 5000**

**R_d 3**

**Mem_flu 6**

**P0 3**

**P1 60**

**P2 100**

**P3 100**

**PB 200**

simulation_1_Superfluidity.m

clc;clear;

Parameter = importdata('simulation_1_Superfluidity.txt');

% s:size of simulation area (shape:square,unit:nm)

% s_t:Simulation step time (us)

% t:Simulation time (us)

% R_d:interac distance of proteins

% Mem_flu:Fluidity of cell membrane (influence interaction times)

% P0:ligand generate num (per step)

% P1:receptor protein num (constant)

% P2:First recruited protein num (constant)

% P3:Second recruited protein num (constant)

% PB:Depolymerization protein num (constant)

s=Parameter.data(1);s_t=Parameter.data(2);t=Parameter.data(3);R_d=Parameter.data(4);Mem_flu=Parameter.data(5);

P0=Parameter.data(6);P1=Parameter.data(7);P2=Parameter.data(8);P3=Parameter.data(9);PB=Parameter.data(10);

%Conp_free repersent legend protein,Conp_1 repersent receptor,Conp_2 repersent First recruited protein,

%Conp_3 repersent Second recruited protein, Conp_break repersent arrestin

%MED-1,2,3 repersent compound 1,2,3

t=t*Mem_flu/3;Conp_1=Protein_G(s,P1);Conp_2=Protein_G(s,P2);Conp_3=Protein_G(s,P3);Conp_break=Protein_G(s,PB);

MED_1=[];MED_2=[];MED_3=[];MED_M_1=[];MED_M_2=[];Conp_repl=[];Count=zeros(1,t);Count_w=zeros(1,t);

%%

for i=1:t

Conp_free=Protein_G(s,3);

[~,Conp_1,MED_M_1]=Protein_C(Conp_free,Conp_1,s,R_d);

MED_1=[MED_1,MED_M_1];

T1(i)=length(MED_1);

Conp_1=Protein_M(Conp_1,Mem_flu,s_t,s);

if ~isempty (MED_1)

MED_1=Protein_M(MED_1,Mem_flu,s_t,s);

[Conp_2,MED_1,MED_M_2]=Protein_C(Conp_2,MED_1,s,R_d);

Conp_2=Protein_M(Conp_2,Mem_flu,s_t,s);

MED_2=[MED_2,MED_M_2];

T2(i)=length(MED_2);

if ~isempty (MED_2)

MED_2=Protein_M(MED_2,0.8*Mem_flu,s_t,s);

[Conp_3,MED_2,MED_M_3]=Protein_C(Conp_3,MED_2,s,R_d);

MED_3=[MED_3,MED_M_3];

T3(i)=length(MED_3);

if ~isempty (MED_3)

MED_3=Protein_M(MED_3,0.4*Mem_flu,s_t,s);

[~,MED_3,Conp_repl]=Protein_C(Conp_break,MED_3,s,R_d);

Conp_break=Protein_M(Conp_break,Mem_flu,s_t,s);

else

Conp_repl=[];

end

else

T3(i)=length(MED_3);

end

else

T2(i)=length(MED_2);

T3(i)=length(MED_3);

end

if ~isempty(MED_M_1)

Count(i+1)=Count(i)+length(MED_M_1(1,:));

else

Count(i+1)=Count(i);

end

if ~isempty(MED_3)

Count_w(i+1)=Count_w(i)+length(MED_3(1,:));

else

Count_w(i+1)=Count_w(i);

end

if i<=50

eval(['Sp1_',num2str(i),'=Conp_repl',';']);

eval(['Sp2_',num2str(i),'=Conp_repl',';']);

eval(['Sp3_',num2str(i),'=Conp_repl',';']);

for gn=1:i

eval(['Sp1_',num2str(gn),'=Protein_M(Sp1_',num2str(gn),',2*Mem_flu,s_t,s)',';']);

eval(['Sp2_',num2str(gn),'=Protein_M(Sp1_',num2str(gn),',2*Mem_flu,s_t,s)',';']);

eval(['Sp3_',num2str(gn),'=Protein_M(Sp1_',num2str(gn),',2*Mem_flu,s_t,s)',';']);

end

else

kk=mod(i,50);

if kk==0

kk=50;

end

eval(['Conp_1=[Conp_1,Sp1_',num2str(kk),']',';']);

eval(['Conp_2=[Conp_2,Sp2_',num2str(kk),']',';']);

eval(['Conp_3=[Conp_2,Sp3_',num2str(kk),']',';']);

eval(['Sp1_',num2str(kk),'=Conp_repl',';']);

eval(['Sp2_',num2str(kk),'=Conp_repl',';']);

eval(['Sp3_',num2str(kk),'=Conp_repl',';']);

for gn=1:50

eval(['Sp1_',num2str(gn),'=Protein_M(Sp1_',num2str(gn),',2*Mem_flu,s_t,s)',';']);

eval(['Sp2_',num2str(gn),'=Protein_M(Sp1_',num2str(gn),',2*Mem_flu,s_t,s)',';']);

eval(['Sp3_',num2str(gn),'=Protein_M(Sp1_',num2str(gn),',2*Mem_flu,s_t,s)',';']);

end

end

end

if length(Count)>t

Count(1)=[];

Count_w(1)=[];

end

Count_w_afit=Count_w/100;

for i=1:(t/(Mem_flu/3));

N1(i)=T1((Mem_flu/3)*i);

N2(i)=T2((Mem_flu/3)*i);

N3(i)=T3((Mem_flu/3)*i);

Count_c_N(i)=Count((Mem_flu/3)*i);

Count_w_afit_N(i)=Count_w_afit((Mem_flu/3)*i);

end

T1=N1;T2=N2;T3=N3;Count=Count_c_N;Count_w_afit=Count_w_afit_N;

%%

%

% save E:\Desktop\simulation\SIm2\SB_2\N1.txt N1 -ascii -tabs

% save E:\Desktop\simulation\SIm2\SB_2\N2.txt N2 -ascii -tabs

% save E:\Desktop\simulation\SIm2\SB_2\N3.txt N3 -ascii -tabs

% save E:\Desktop\simulation\SIm2\SB_2\Count_c_N.txt Count_c_N -ascii -tabs

% save E:\Desktop\simulation\SIm2\SB_2\Count_w_afit_N.txt Count_w_afit_N -ascii -tabs

figure

subplot(3,1,1)

plot(T1)

xlabel('Simulation time (us)')

title('Protein complex 1 (T1)')

subplot(3,1,2)

plot(T2)

xlabel('Simulation time (us)')

title('Protein complex 2 (T2)')

subplot(3,1,3)

plot(T3)

xlabel('Simulation time (us)')

title('Protein complex 2 (T3)')

figure

subplot(2,1,1)

plot(Count)

xlabel('Simulation time (us)')

title('Receptor protein usage')

subplot(2,1,2)

plot(Count_w_afit)

xlabel('Simulation time (us)')

title('Signal output strength')

simulation_2_Normal.txt

**s 200**

**s_t 1**

**t_g 50**

**R_d 3**

**Mem_flu 3**

**ST_PO 2**

**AC_PO 60**

**P1 60**

**P2 100**

**P3 100**

**PB 200**

simulation_2_Normal.m

clc;clear;

Parameter = importdata('simulation_2_Normal.txt');

s=Parameter.data(1);s_t=Parameter.data(2);t_g=Parameter.data(3);R_d=Parameter.data(4);Mem_flu=Parameter.data(5);

ST_P0=Parameter.data(6);AC_P0=Parameter.data(7);P1=Parameter.data(8);P2=Parameter.data(9);P3=Parameter.data(10);PB=Parameter.data(11);

% s:size of simulation area (shape:square,unit:nm)

% s_t:Simulation step time (us)

% t_g:Simulation period (us)

% R_d:interac distance of proteins

% Mem_flu:Fluidity of cell membrane (influence interaction times)

% ST_P0:ligand generate num in Resting state (per step)

% AC_P0:ligand generate num in Stimulus state (per step)

% P1:receptor protein num (constant)

% P2:First recruited protein num (constant)

% P3:Second recruited protein num (constant)

% PB:Depolymerization protein num (constant)

%Conp_free repersent legend protein,Conp_1 repersent receptor,Conp_2 repersent First recruited protein,

%Conp_3 repersent Second recruited protein, Conp_break repersent arrestin

%MED-1,2,3 repersent compound 1,2,3

g=t_g*(Mem_flu/3);t=12*t_g;Conp_1=Protein_G(s,P1);Conp_2=Protein_G(s,P2);Conp_3=Protein_G(s,P3);Conp_break=Protein_G(s,PB);

MED_1=[];MED_2=[];MED_3=[];MED_M_1=[];MED_M_2=[];Conp_repl=[];Count=zeros(1,t);Count_w=zeros(1,t);

%Generate ligand protein sequence,seq1 represent Resting state,seq2;represent Stimulus state;

seq1=ceil(ST_P0*rand(2*g,1));

seq2=AC_P0*ones(2*g,1)+(AC_P0/10)*ceil(wgn(2*g,1,10));

seq=[seq1;seq2;seq1;seq1;seq2;seq1];

%%

for i=1:t

Conp_free=Protein_G(s,seq(i));

[~,Conp_1,MED_M_1]=Protein_C(Conp_free,Conp_1,s,R_d);

MED_1=[MED_1,MED_M_1];

T1(i)=length(MED_1);

Conp_1=Protein_M(Conp_1,Mem_flu,s_t,s);

if ~isempty (MED_1)

MED_1=Protein_M(MED_1,Mem_flu,s_t,s);

[Conp_2,MED_1,MED_M_2]=Protein_C(Conp_2,MED_1,s,R_d);

Conp_2=Protein_M(Conp_2,Mem_flu,s_t,s);

MED_2=[MED_2,MED_M_2];

T2(i)=length(MED_2);

if ~isempty (MED_2)

MED_2=Protein_M(MED_2,0.8*Mem_flu,s_t,s);

[Conp_3,MED_2,MED_M_3]=Protein_C(Conp_3,MED_2,s,R_d);

MED_3=[MED_3,MED_M_3];

T3(i)=length(MED_3);

if ~isempty (MED_3)

MED_3=Protein_M(MED_3,0.4*Mem_flu,s_t,s);

[~,MED_3,Conp_repl]=Protein_C(Conp_break,MED_3,s,R_d);

Conp_break=Protein_M(Conp_break,Mem_flu,s_t,s);

else

Conp_repl=[];

end

else

T3(i)=length(MED_3);

end

else

T2(i)=length(MED_2);

T3(i)=length(MED_3);

end

if ~isempty(MED_M_1)

Count(i+1)=Count(i)+length(MED_M_1(1,:));

else

Count(i+1)=Count(i);

end

if ~isempty(MED_3)

Count_w(i+1)=Count_w(i)+length(MED_3(1,:));

else

Count_w(i+1)=Count_w(i);

end

Conp_free=Protein_G(s,10);

if i<=50

eval(['Sp1_',num2str(i),'=Conp_repl',';']);

eval(['Sp2_',num2str(i),'=Conp_repl',';']);

eval(['Sp3_',num2str(i),'=Conp_repl',';']);

for gn=1:i

eval(['Sp1_',num2str(gn),'=Protein_M(Sp1_',num2str(gn),',2*Mem_flu,s_t,s)',';']);

eval(['Sp2_',num2str(gn),'=Protein_M(Sp1_',num2str(gn),',2*Mem_flu,s_t,s)',';']);

eval(['Sp3_',num2str(gn),'=Protein_M(Sp1_',num2str(gn),',2*Mem_flu,s_t,s)',';']);

end

else

kk=mod(i,50);

if kk==0

kk=50;

end

eval(['Conp_1=[Conp_1,Sp1_',num2str(kk),']',';']);

eval(['Conp_2=[Conp_2,Sp2_',num2str(kk),']',';']);

eval(['Conp_3=[Conp_2,Sp3_',num2str(kk),']',';']);

eval(['Sp1_',num2str(kk),'=Conp_repl',';']);

eval(['Sp2_',num2str(kk),'=Conp_repl',';']);

eval(['Sp3_',num2str(kk),'=Conp_repl',';']);

for gn=1:50

eval(['Sp1_',num2str(gn),'=Protein_M(Sp1_',num2str(gn),',2*Mem_flu,s_t,s)',';']);

eval(['Sp2_',num2str(gn),'=Protein_M(Sp1_',num2str(gn),',2*Mem_flu,s_t,s)',';']);

eval(['Sp3_',num2str(gn),'=Protein_M(Sp1_',num2str(gn),',2*Mem_flu,s_t,s)',';']);

end

end

end

if length(Count)>t

Count(1)=[];

Count_w(1)=[];

end

Count_w_afit=Count_w/100;

%%

% save E:\Desktop\simulation\SIm2\pulse_L\T1.txt -ascii T1 -ascii -tabs

% save E:\Desktop\simulation\SIm2\pulse_L\T2.txt -ascii T2 -ascii -tabs

% save E:\Desktop\simulation\SIm2\pulse_L\T3.txt -ascii T3 -ascii -tabs

% save E:\Desktop\simulation\SIm2\pulse_L\Count.txt -ascii Count -ascii -tabs

% save E:\Desktop\simulation\SIm2\pulse_L\Count_w_afit_N.txt -ascii Count_w_afit -ascii -tabs

figure

subplot(3,1,1)

plot(T1)

xlabel('Simulation time (us)')

title('Protein complex 1 (T1)')

subplot(3,1,2)

plot(T2)

xlabel('Simulation time (us)')

title('Protein complex 2 (T2)')

subplot(3,1,3)

plot(T3)

xlabel('Simulation time (us)')

title('Protein complex 2 (T3)')

figure

subplot(2,1,1)

plot(Count)

xlabel('Simulation time (us)')

title('Receptor protein usage')

subplot(2,1,2)

plot(Count_w_afit)

xlabel('Simulation time (us)')

title('Signal output strength')

simulation_2_Superfluidity.txt

**s 200**

**s_t 1**

**t_g 50**

**R_d 3**

**Mem_flu 6**

**ST_PO 2**

**AC_PO 60**

**P1 60**

**P2 100**

**P3 100**

**PB 200**

simulation_2_Superfluidity.m

clc;clear;

Parameter = importdata('simulation_2_Superfluidity.txt');

s=Parameter.data(1);s_t=Parameter.data(2);t_g=Parameter.data(3);R_d=Parameter.data(4);Mem_flu=Parameter.data(5);

ST_P0=Parameter.data(6);AC_P0=Parameter.data(7);P1=Parameter.data(8);P2=Parameter.data(9);P3=Parameter.data(10);PB=Parameter.data(11);

% s:size of simulation area (shape:square,unit:nm)

% s_t:Simulation step time (us)

% t_g:Simulation period (us)

% R_d:interac distance of proteins

% Mem_flu:Fluidity of cell membrane (influence interaction times)

% ST_P0:ligand generate num in Resting state (per step)

% AC_P0:ligand generate num in Stimulus state (per step)

% P1:receptor protein num (constant)

% P2:First recruited protein num (constant)

% P3:Second recruited protein num (constant)

% PB:Depolymerization protein num (constant)

%Conp_free repersent legend protein,Conp_1 repersent receptor,Conp_2 repersent First recruited protein,

%Conp_3 repersent Second recruited protein, Conp_break repersent arrestin

%MED-1,2,3 repersent compound 1,2,3

g=t_g*(Mem_flu/3);t=12*g;Conp_1=Protein_G(s,P1);Conp_2=Protein_G(s,P2);Conp_3=Protein_G(s,P3);Conp_break=Protein_G(s,PB);

MED_1=[];MED_2=[];MED_3=[];MED_M_1=[];MED_M_2=[];Conp_repl=[];Count=zeros(1,t);Count_w=zeros(1,t);

%Generate ligand protein sequence,seq1 represent Resting state,seq2;represent Stimulus state;

seq1=ceil(ST_P0*rand(2*g,1));

seq2=AC_P0*ones(2*g,1)+(AC_P0/10)*ceil(wgn(2*g,1,10));

seq=[seq1;seq2;seq1;seq1;seq2;seq1];

%%

for i=1:t

Conp_free=Protein_G(s,seq(i));

[~,Conp_1,MED_M_1]=Protein_C(Conp_free,Conp_1,s,R_d);

MED_1=[MED_1,MED_M_1];

T1(i)=length(MED_1);

Conp_1=Protein_M(Conp_1,Mem_flu,s_t,s);

if ~isempty (MED_1)

MED_1=Protein_M(MED_1,Mem_flu,s_t,s);

[Conp_2,MED_1,MED_M_2]=Protein_C(Conp_2,MED_1,s,R_d);

Conp_2=Protein_M(Conp_2,Mem_flu,s_t,s);

MED_2=[MED_2,MED_M_2];

T2(i)=length(MED_2);

if ~isempty (MED_2)

MED_2=Protein_M(MED_2,0.8*Mem_flu,s_t,s);

[Conp_3,MED_2,MED_M_3]=Protein_C(Conp_3,MED_2,s,R_d);

MED_3=[MED_3,MED_M_3];

T3(i)=length(MED_3);

if ~isempty (MED_3)

MED_3=Protein_M(MED_3,0.4*Mem_flu,s_t,s);

[~,MED_3,Conp_repl]=Protein_C(Conp_break,MED_3,s,R_d);

Conp_break=Protein_M(Conp_break,Mem_flu,s_t,s);

else

Conp_repl=[];

end

else

T3(i)=length(MED_3);

end

else

T2(i)=length(MED_2);

T3(i)=length(MED_3);

end

if ~isempty(MED_M_1)

Count(i+1)=Count(i)+length(MED_M_1(1,:));

else

Count(i+1)=Count(i);

end

if ~isempty(MED_3)

Count_w(i+1)=Count_w(i)+length(MED_3(1,:));

else

Count_w(i+1)=Count_w(i);

end

Conp_free=Protein_G(s,10);

% Conp_free=Protein_G(s,50);

if i<=50

eval(['Sp1_',num2str(i),'=Conp_repl',';']);

eval(['Sp2_',num2str(i),'=Conp_repl',';']);

eval(['Sp3_',num2str(i),'=Conp_repl',';']);

for gn=1:i

eval(['Sp1_',num2str(gn),'=Protein_M(Sp1_',num2str(gn),',2*Mem_flu,s_t,s)',';']);

eval(['Sp2_',num2str(gn),'=Protein_M(Sp1_',num2str(gn),',2*Mem_flu,s_t,s)',';']);

eval(['Sp3_',num2str(gn),'=Protein_M(Sp1_',num2str(gn),',2*Mem_flu,s_t,s)',';']);

end

else

kk=mod(i,50);

if kk==0

kk=50;

end

eval(['Conp_1=[Conp_1,Sp1_',num2str(kk),']',';']);

eval(['Conp_2=[Conp_2,Sp2_',num2str(kk),']',';']);

eval(['Conp_3=[Conp_2,Sp3_',num2str(kk),']',';']);

eval(['Sp1_',num2str(kk),'=Conp_repl',';']);

eval(['Sp2_',num2str(kk),'=Conp_repl',';']);

eval(['Sp3_',num2str(kk),'=Conp_repl',';']);

for gn=1:50

eval(['Sp1_',num2str(gn),'=Protein_M(Sp1_',num2str(gn),',2*Mem_flu,s_t,s)',';']);

eval(['Sp2_',num2str(gn),'=Protein_M(Sp1_',num2str(gn),',2*Mem_flu,s_t,s)',';']);

eval(['Sp3_',num2str(gn),'=Protein_M(Sp1_',num2str(gn),',2*Mem_flu,s_t,s)',';']);

end

end

end

if length(Count)>t

Count(1)=[];

Count_w(1)=[];

end

Count_w_afit=Count_w/100;

for i=1:(t/(Mem_flu/3));

N1(i)=T1((Mem_flu/3)*i);

N2(i)=T2((Mem_flu/3)*i);

N3(i)=T3((Mem_flu/3)*i);

Count_c_N(i)=Count((Mem_flu/3)*i);

Count_w_afit_N(i)=Count_w_afit((Mem_flu/3)*i);

end

T1=N1;T2=N2;T3=N3;Count=Count_c_N;Count_w_afit=Count_w_afit_N;

%%

% save E:\Desktop\simulation\SIm2\pulse_H\N1.txt -ascii N1 -ascii -tabs

% save E:\Desktop\simulation\SIm2\pulse_H\N2.txt -ascii N2 -ascii -tabs

% save E:\Desktop\simulation\SIm2\pulse_H\N3.txt -ascii N3 -ascii -tabs

% save E:\Desktop\simulation\SIm2\pulse_H\Count_c_N.txt -ascii Count_c_N -ascii -tabs

% save E:\Desktop\simulation\SIm2\pulse_H\Count_w_afit_N.txt -ascii Count_w_afit_N -ascii -tabs

%

figure

subplot(3,1,1)

plot(T1)

xlabel('Simulation time (us)')

title('Protein complex 1 (T1)')

subplot(3,1,2)

plot(T2)

xlabel('Simulation time (us)')

title('Protein complex 2 (T2)')

subplot(3,1,3)

plot(T3)

xlabel('Simulation time (us)')

title('Protein complex 2 (T3)')

figure

subplot(2,1,1)

plot(Count)

xlabel('Simulation time (us)')

title('Receptor protein usage')

subplot(2,1,2)

plot(Count_w_afit)

xlabel('Simulation time (us)')

title('Signal output strength')
