# Supplementary figures and images for: Bioglass could increase cell membrane fluidity with ion products to develop its bioactivity
Source: Cell Prolif. 2020 Oct 11;53(11):e12906. doi: 10.1111/cpr.12906 (PMC7653244; doi:10.1111/cpr.12906)

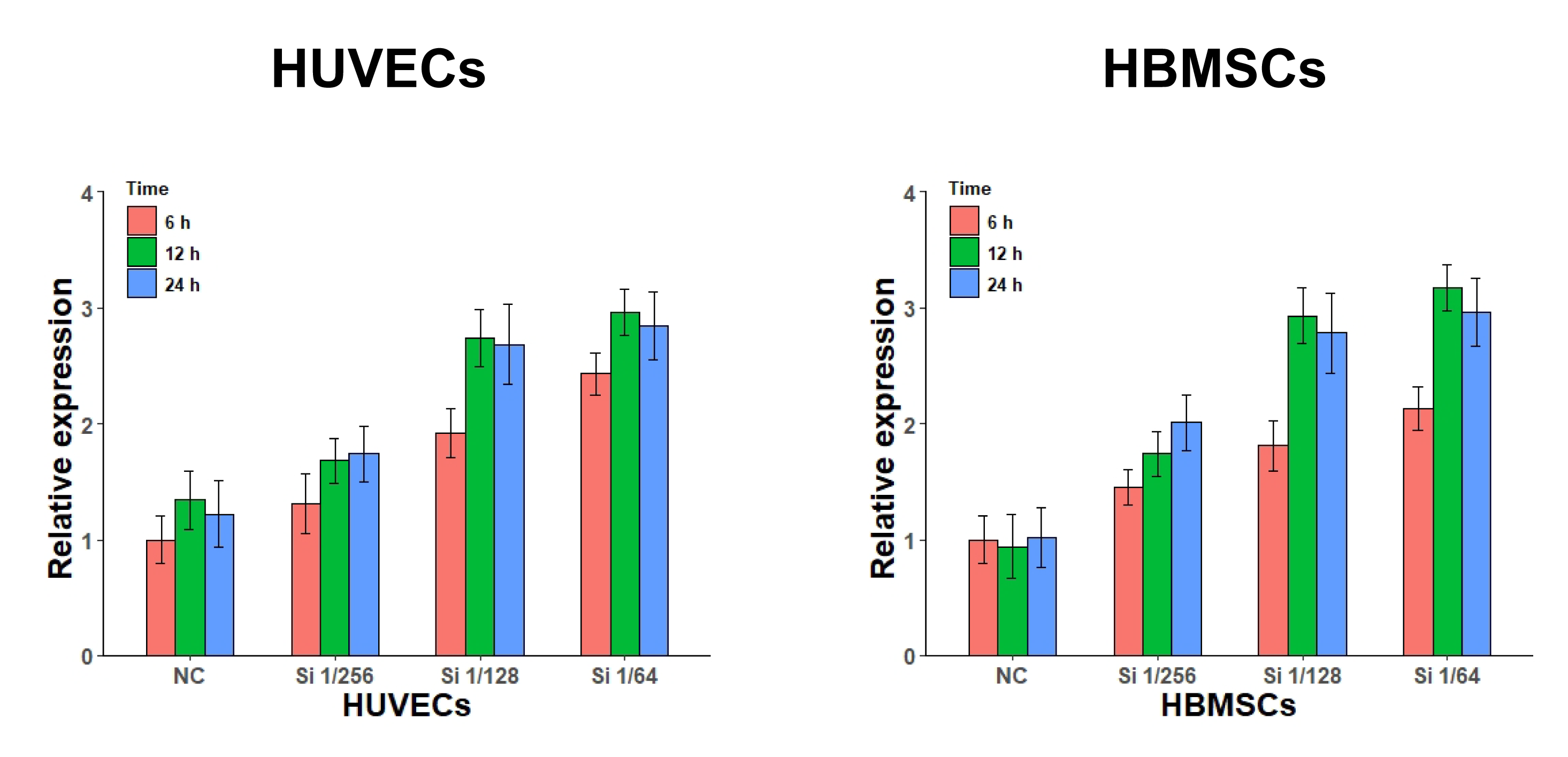

Supplement: Supplementary file 1 — Supplementary Material [file CPR-53-e12906-s001.zip › cpr12906-sup-0002-FigS1.tif]

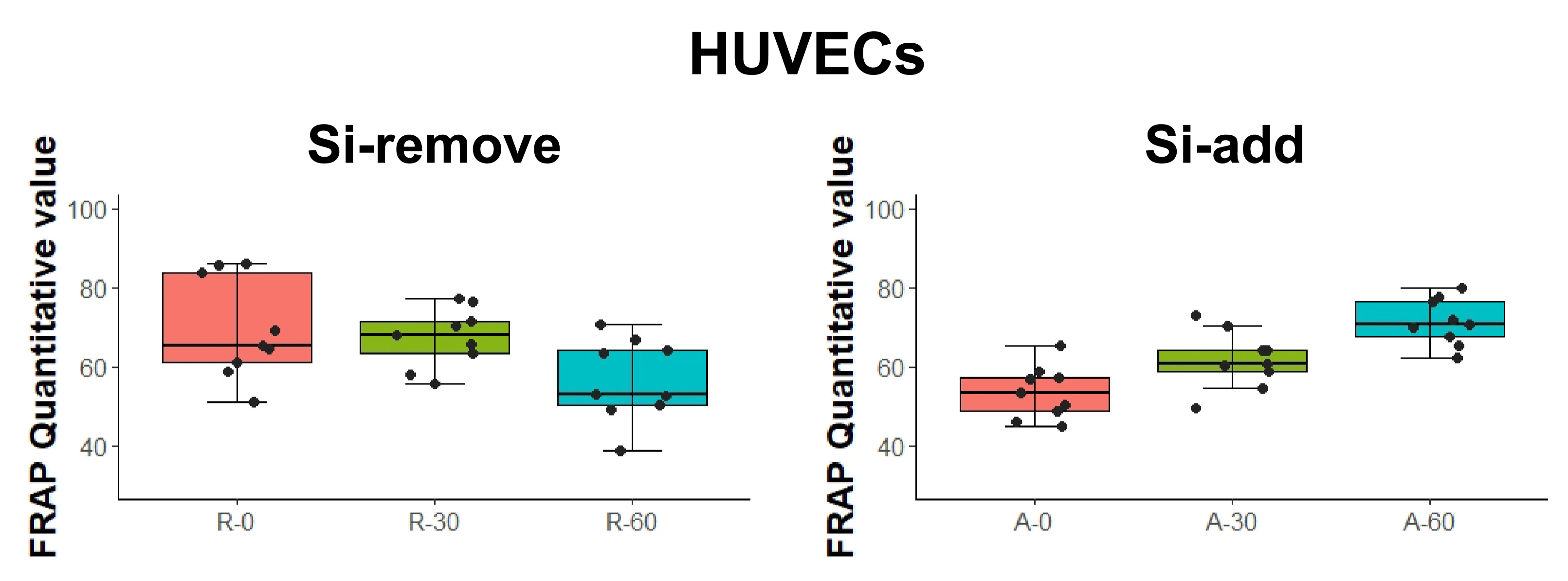

Supplement: Supplementary file 1 — Supplementary Material [file CPR-53-e12906-s001.zip › cpr12906-sup-0003-FigS2.tif]

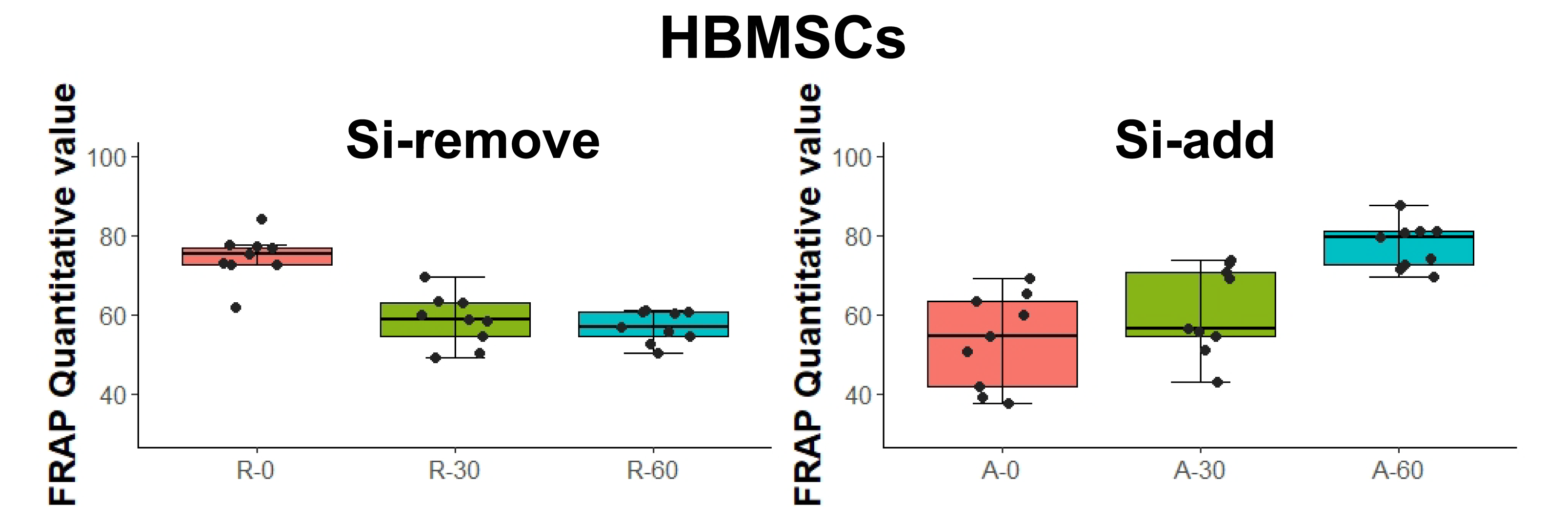

Supplement: Supplementary file 1 — Supplementary Material [file CPR-53-e12906-s001.zip › cpr12906-sup-0004-FigS3.tif]
